# An Arabidopsis mutant impaired in intracellular calcium elevation is sensitive to biotic and abiotic stress

**DOI:** 10.1186/1471-2229-14-162

**Published:** 2014-06-11

**Authors:** Joy Michal Johnson, Michael Reichelt, Jyothilakshmi Vadassery, Jonathan Gershenzon, Ralf Oelmüller

**Affiliations:** 1 Institute of General Botany and Plant Physiology, Friedrich-Schiller-University Jena, Dornburger Str. 159, 07743 Jena, Germany; 2 Max Planck Institute for Chemical Ecology, Beutenberg Campus, Hans-Knöll-Straße 8, D-07745 Jena, Germany

**Keywords:** Abiotic stress, Biotic stress, *A. brassicae*, Camalexin, Cytosolic calcium elevation, Glucosinolates, Phytohormones

## Abstract

**Background:**

Ca^2+^, a versatile intracellular second messenger in various signaling pathways, initiates many responses involved in growth, defense and tolerance to biotic and abiotic stress. Endogenous and exogenous signals induce cytoplasmic Ca^2+^ ([Ca^2+^]_cyt_) elevation, which are responsible for the appropriate downstream responses.

**Results:**

Here we report on an ethyl-methane sulfonate-mediated Arabidopsis mutant that fails to induce [Ca^2+^]_cyt_ elevation in response to exudate preparations from the pathogenic mibrobes *Alternaria brassicae*, *Rhizoctonia solani*, *Phytophthora parasitica* var. *nicotianae* and *Agrobacterium tumefaciens*. The ***cy****toplasmic ****Ca***^***2****+*^*elevation ****m****utant1* (*cycam1*) is susceptible to infections by *A. brassicae*, its toxin preparation and sensitive to abiotic stress such as drought and salt. It accumulates high levels of reactive oxygen species and contains elevated salicylic acid, abscisic acid and bioactive jasmonic acid iso-leucine levels. Reactive oxygen species- and phytohormone-related genes are higher in *A. brassicae*-treated wild-type and mutant seedlings. Depending on the analysed response, the elevated levels of defense-related compounds are either caused by the *cycam* mutation and are promoted by the pathogen, or they are mainly due to the pathogen infection or application of pathogen-associated molecular patterns. Furthermore, *cycam1* shows altered responses to abscisic acid treatments: the hormone inhibits germination and growth of the mutant.

**Conclusions:**

We isolated an Arabidopsis mutant which fails to induce [Ca^2+^]_cyt_ elevation in response to exudate preparations from various microbes. The higher susceptibility of the mutant to pathogen infections correlates with the higher accumulation of defense-related compounds, such as phytohormones, reactive oxygen-species, defense-related mRNA levels and secondary metabolites. Therefore, CYCAM1 couples [Ca^2+^]_cyt_ elevation to biotic, abiotic and oxidative stress responses.

## Background

Plants have evolved effective mechanisms to perceive, transduce and respond to a wide variety of biotic and abiotic signals by modulating cytosolic Ca^2+^ levels ([Ca^2+^]_cyt_) (c.f. [1-7]). Ca^2+^ is a tightly regulated ion within cellular compartments, and the spatial and temporal control of its concentration makes it a versatile signalling component in plants [5,8]. Under resting conditions, the [Ca^2+^]_cyt_ is maintained below 100 nM, 10^4^ times less than in the apoplastic fluid and 10^4^ to 10^5^ times less than in vacuoles, endoplasmic reticulum and chloroplasts [2,5]. The Ca^2+^ signaling system is composed of a receptor, a system for generating the transient increase in [Ca^2+^]_cyt_ through Ca^2+^-pumps and -channels in response to a stimulus, recognition of the specific Ca^2+^-signature by sensor proteins and transduction of the information to targets, and cellular systems responsible for returning [Ca^2+^]_cyt_ to its pre-stimulus level [9,10]. In plants, increase in [Ca^2+^]_cyt_ arises from the influx of Ca^2+^ from the apoplast and/or from internal stores through specific channels like cyclic nucleotide gated channels, glutamate receptor channels or two pore Ca^2+^ channels [1,9-11]. H^+^/Ca^2+^ antiporters and Ca^2+^-ATPases pump the Ca^2+^ ions back into the apoplast and/or intracellular stores once the receptor is no longer activated by ligand binding [10].

[Ca^2+^]_cyt_ elevation is one of the earliest physiological events in root and leaf cells in response to pathogenic stimuli. Upon perception of signals from pathogenic fungi or/and their pathogen-associated molecular patterns (PAMPs), [Ca^2+^]_cyt_ levels transiently increase in the host cells within seconds [4,12-15]. Plants discriminate both the nature and strength of these stimuli to mount an appropriate rapid adaptive response for their survival [16]. Recognition and perception of fungal pathogens via their PAMPs or effectors induces [Ca^2+^]_cyt_ elevation which leads to the activation of defence-signalling cascades against the attempted pathogen invasion [12,17,18].

Here, we report on an Arabidopsis mutant which was isolated due to its failure to induce [Ca^2+^]_cyt_ elevation in response to exudate components from *Alternaria brassicae* (Berk.) Sacc. *A. brassicae* is a necrotrophic deuteromycete fungus which causes black spot disease in crucifers including *A. thaliana*. It is a seed-, air- and soil-borne fungus that penetrates through all plant parts and causes lesions on leaves, stems, siliques and roots [19]. The disease progression ultimately results in plant death, mostly caused by host-specific toxins (Tox) [19-23]. These are low molecular weight secondary metabolites of different chemical classes which can be isolated from liquid cultures or germinating spores [22-25]. The two well known phytotoxins destruxin B and sirodesmin PL from *A. brassicae* induce phytoalexin and camalexin biosynthesis in crucifers [23,26].

We demonstrate that besides these Toxs, non-toxic low molecular weight exudates components from *A. brassicae* also induce [Ca^2+^]_cyt_ elevation in Arabidopsis stably expressing the Ca^2+^ reporter protein aequorin. We have isolated and characterized a ***cy****tosolic ****ca****lcium elevation ****m****utant1* (*cycam1*) which does not induce [Ca^2+^]_cyt_ elevation in response to the non-toxic exudate components. Further characterization of *cycam1* demonstrated that it also fails to induce [Ca^2+^]_cyt_ elevation in response to exudate preparations from *Rhizoctonia solani*, *Phytophthora parasitica* var. *nicotianae* and *Agrobacterium tumefaciens*. The mutant is susceptible to infection by *A. brassicae* and sensitive to abscisic acid (ABA), drought and salt stress. Thus, the mutated gene couples [Ca^2+^]_cyt_ elevation to biotic and abiotic stress responses.

## Results

### Exudate components from *A. brassicae* induce [Ca^2+^]_cyt_ elevation in *Arabidopsis* roots

**Figure 1:**
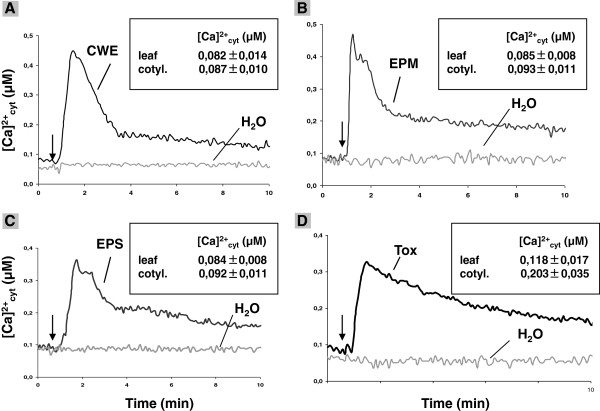
***A. brassicae*-CWE, -EPM, -EPS and -Tox induce [Ca**^**2+**^**]**_**cyt **_**elevation in *A. thaliana *seedlings expressing cytosolic aequorin.** Roots of 18-day old pMAQ2 in Col-0 seedlings were dissected and incubated overnight in 7.5 μM coelenterazine. The roots were challenged with 50 μl of the CWE **(A)**, EPM **(B)**, EPS **(C)** or Tox **(D)** preparations. [Ca^2+^]_cyt_ level was calculated from the relative light unit (RLU) at 5 s integration time for 10 min. The arrow indicates the time (60 s) of addition of the stimuli/water. The inset shows the mean peak values ± SEs of [Ca^2+^]_cyt_ elevation in leaves and cotyledons (cotyl.) with the same dose of stimuli. Sterile water was used as control and gave background readings. All curves and values represent average of five independent experiments with eight replications in each experiment.

Under resting conditions, 18 d-old transgenic apoaequorin-carrying *A. thaliana* roots in the Col-0 background (pMAQ2) [27,28] gave [Ca^2+^]_cyt_ values of 70 ± 0,6 nM (*n* = 16). A rapid and transient increase in the [Ca^2+^]_cyt_ concentration is observed 40 sec after the application of a cell wall extract (CWE), a water-diffusible exudate preparation from mycelia (EPM), germinating spores (EPS) or a Tox preparation from *A. brassica*e to the roots (Figure 1). Discharge at the end of the experiment demonstrates that less than 5% of the reconstituted aequorin was consumed after the stimuli, which ensures that the amount of aequorin in the sample is not limiting for the Ca^2+^ signal [16]. After a lag phase of 15 – 20 sec, the levels of [Ca^2+^]_cyt_ begin to rise and reach a peak of ~ 300 – 400 nM after 40 to 70 sec (Figure 1). Subsequently the Ca^2+^ levels steadily decreased. No [Ca^2+^]_cyt_ elevation is observed with the water control treatment (Figure 1) and barely any [Ca^2+^]_cyt_ elevation is observed in response to the CWE, EPM and EPS in the cotyledons of 18 d-old seedlings, while the Tox preparation induces [Ca^2+^]_cyt_ elevation in the cotyledons although at lower rates than in the roots (Figure 1, insets). For all stimuli, the magnitudes of the [Ca^2+^]_cyt_ responses are dose-dependent (Additional file 1: Figure S1).

The *A. brassicae* exudates and Tox preparations showed very similar [Ca^2+^]_cyt_ elevation kinetics which did not change after heat treatment (20 min at 121°C by autoclaving) indicating that the components are thermostable (Additional file 1: Figure S2). After ethyl acetate extraction the Ca^2+^ activity in the aequous phase was comparable to the activity in the starting fraction, while barely any activity was detectable after evaporating the ethyl acetate and resolving the residual material in an equal volume of distilled water. This indicates that most of the activity remained in the aqueous phase. Similar results were obtained after extracting the CWE, EPM and EPS preparations with methanol, whereas extraction of the Tox preparation with methanol resulted in a supernatant and precipitate fraction which showed [Ca^2+^]_cyt_ inducing activities (Additional file 1: Figure S2). This suggests that the [Ca^2+^]_cyt_ activity induced by the Tox preparation is different from those induced by the three other preparations. Size separation of the fungal components demonstrates that all compounds are < 3 kDa (Additional file 1: Figure S2).

### A Ca^2+^-based screen to isolate mutants defective in [Ca^2+^]_cyt_ elevation to the CWE

**Figure 2:**
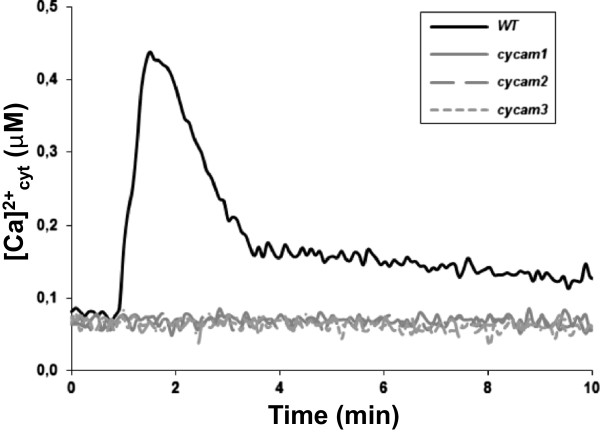
**Mutants which do not respond to *A. brassicae *CWE.** 18-day old M2 seedlings from the individual M1 plants were used for the mutant screening. About 70% of the roots from the individual M2 seedlings was dissected and incubated in 7.5 μM coelenterazine overnight and challenged with the CWE. *cycam1*, *cycam2* and *cycam3* did not respond to the CWE.

96-well plates in combination with a plate-reader luminometer equipped with an automatic injection system were used to screen for Arabidopsis mutants which do not show [Ca^2+^]_cyt_ elevation in response to the *A. brassicae* CWE. The screen was performed with roots from individual 18-day-old M_2_ seedlings, after ethyl methane sulfonate (EMS) mutagenesis of transgenic apoaequorin-carrying M_1_ seeds in the Col-0 background [27,28]. After recording the background [Ca^2+^]_cyt_ level for 1 min, the response to the CWE was measured for 10 min. Roots which did not respond to the stimulus were used for the total discharge reaction to ensure that the lack of [Ca^2+^]_cyt_ elevation is not caused by a mutation in the apoaequorin gene. Screening of approximately 150.000 individual M_2_ plants identified 12 mutants which completely failed to induce [Ca^2+^]_cyt_ elevation in response to the CWE; they were named ***cy****toplasmic ****ca****lcium elevation ****m****utants* (*cycam*) (Figure 2). They were transferred to soil to obtain M_3_ and M_4_ seeds. Three putative mutants did not survive in soil. For the other lines, the phenotype was confirmed with the M_3_ and M_4_ lines. None of them showed a visible phenotype under our growth conditions when compared to WT. Genetic analyses of crosses uncovered that four *cycam* mutants were allelic. Two of them, *cycam1-1* and *cycam1-2*, were randomly chosen and used for further analyses. When *cycam1-1* and *cycam1-2* were backcrossed to WT (Col-0) or WT (La), [Ca^2+^]_cyt_ elevation to the CWE was restored in ~25% of F_2_ progenies, indicating that the mutations are recessive.

### *cycam1* does not respond to the EPM and EPS, but responded to the Tox preparation.

**Figure 3:**
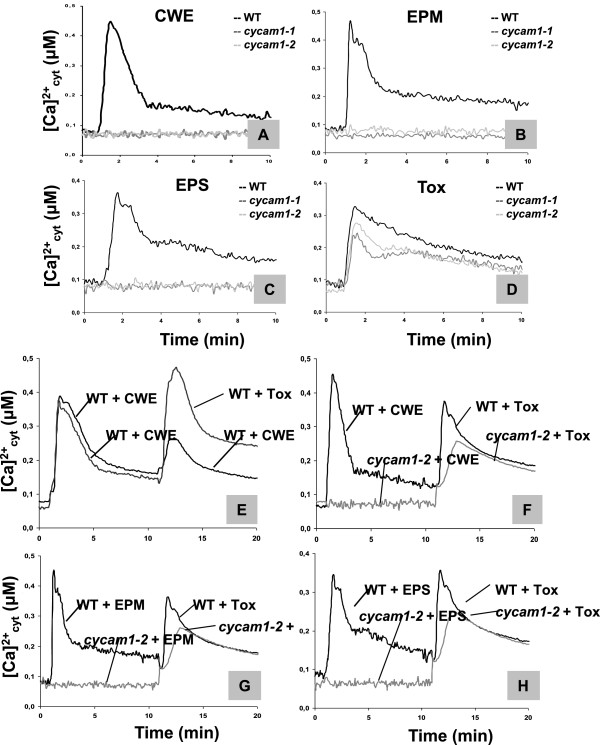
**Response of WT and *cycam1 *to different *A. brassicae*-derived stimuli.** The roots of 18-day old WT and *cycam1* seedlings were dissected and incubated overnight in 7.5 μM coelenterazine. The roots of WT, *cycam1-1* and *cycam1-2* were challenged with 50 μl of CWE **(A)**, EPM **(B)**, EPS **(C)** or Tox **(D)**. The mutants did not respond to the CWE, EPM and EPS **(A-C)** but responded to the Tox preparation **(D)**. pMAQ2 in Col-0 seedlings served as control. Refractory behavior of the fungal stimuli to [Ca^2+^]_cyt_ changes in WT and *cycam1-2* were determined by competition assays **(E-H)**. WT and mutant roots were first treated either with the CWE, the EPM or EPS and subsequently 10 min later with CWE **(E)** or the Tox **(E-H)**. The CWE-induced [Ca^2+^]_cyt_ change is refractory to consecutive applications of CWE but non-refractory to the second treatment with Tox in WT roots **(E)**. The CWE, EPM or EPS induce [Ca^2+^]_cyt_ changes in WT, but not *cycam1-2* roots, while both WT and mutant responded to subsequent treatment with the Tox **(E-H)**. All curves represent average of four independent experiments with eight replications in each experiment.

The *cycam1-1* and *cycam1-2* roots did not respond to the Ca^2+^-inducing EPM and EPS preparations from *A. brassicae*, but showed a WT response to the Tox preparation (Figure 3A-D). To test whether the [Ca^2+^]_cyt_ responses induced by the CWE, EPM, EPS or the Tox preparations show a refractory behaviour, roots of WT and the two *cycam1* alleles were challenged first with either the CWE, EPM or EPS and subsequently with either the same stimulus or one of the other two stimuli. Ten min after the first stimulus, when the [Ca^2+^]_cyt_ level is on its descent, the second stimulus was applied. Figure 3E demonstrates that a second stimulus with the CWE to WT plants showed a weaker response. The same was observed for EPM or EPS, and any combination of the three stimuli CWE, EPM and EPS (data not shown). The comparable [Ca^2+^]_cyt_ responses with refractory features for the three stimuli indicate that CYCAM1 is involved in all responses. Therefore, the three preparations contain either the same compound or all of them require CYCAM1 for [Ca^2+^]_cyt_ elevation in Arabidopsis roots. When the Tox preparation is applied as a second stimulus, a strong [Ca^2+^]_cyt_ elevation without refractory feature is observed in WT roots, irrespective of whether CWE, EPM or EPS were the first stimuli. The Tox-induced response occurs also in the *cycam1-1* and *cycam1-2* seedlings (Figure 3F-H). Therefore, the Tox preparation-induced [Ca^2+^]_cyt_ response is independent of CYCAM1. Finally, we used flg22 to stimulate [Ca^2+^]_cyt_ elevation in the *cycam1* roots and leaves. No difference to the WT is observed (data not shown).

We applied staurosporine, a protein kinase inhibitor [29-31], to WT roots before the [Ca^2+^]_cyt_ response was induced by the four *A. brassicae*-derived preparations. 5 μM staurosporine was used, because the basal level of [Ca^2+^]_cyt_ and the total aequorin discharge was not changed at this concentration [cf. 32]. Application 1 h prior to the treatment with one of the four Ca^2+^-inducing stimuli significantly reduced [Ca^2+^]_cyt_ elevation (Additional file 1: Table S1). This suggests that the CWE-, EPM-, EPS- and Tox-induced [Ca^2+^]_cyt_ elevation requires kinase activity.

### *cycam1* is also impaired in the [Ca^2+^]_cyt_ response to exudate preparations from other microbes

Since *cycam1* was isolated by a screen in which [Ca^2+^]_cyt_ elevation was impaired in Arabidopsis roots, we further tested CWE and EPM preparations from other microbes with the potential to interact with roots, such as from *Rhizoctonia solani*, a necrotrophic fungus, *Phytophthora parasitica* var. *nicotianae*, a hemibiotrophic oomycete, and *Agrobacterium tumefaciens*, a tumor-inducing bacterium. Interestingly, *cycam1* did not respond to the CWE and EPM preparations from these fungi as well, and less to a CWE from *A. tumefaciens*, even though these preparations induced [Ca^2+^]_cyt_ elevation in WT (Additional file 1: Figure S3A-E). A CWE preparation from the root-colonizing fungus *Mortierella hyalina*[33] induced [Ca^2+^]_cyt_ elevation in the roots of the WT and *cycam1* mutant (data not shown). Therefore, CYCAM1 is involved in [Ca^2+^]_cyt_ elevations in response to different, but not all microbes.

To test whether the [Ca^2+^]_cyt_ responses induced by the CWEs and EPMs from these four microbes show a refractory behaviour, roots of WT and the two *cycam1* alleles were challenged first with the CWE from *A. brassicae* and subsequently with either the CWE or EPM from one of the other microbes. The second stimulus showed always a weaker response. Any combination of the stimuli confirmed that CYCAM1 is involved in all responses.

### *cycam1* is highly susceptible to *A. brassicae* and its Tox preparation

**Figure 4:**
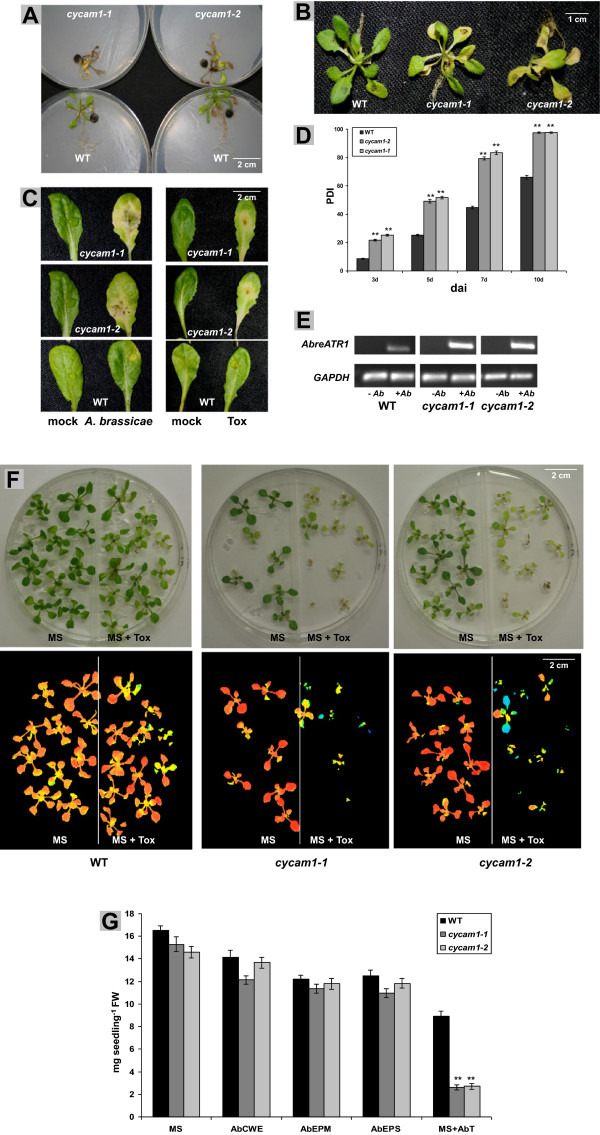
***cycam1-1 *and *cycam1-2 *are highly susceptible to *A. brassicae *and its Tox. (A)** The roots of 14-day old *cycam1* and WT seedlings grown under LD conditions were exposed to a fungal plug for 7 d. **(B)** The leaves of 14-day old *cycam1* and WT seedlings were inoculated with 5 μl spore suspension containing 10^4^-10^5^ cfu ml^−1^ and incubated for 7 d. Detached leaf assays with mature leaves were performed with fungal spores and Tox **(C)**. Mature leaves from 4 week-old *cycam1* and WT plants were dissected, inoculated with 10 μl spore suspension containing 10^4^-10^5^ cfu ml^−1^ or 10 μl Tox preparations and incubated for 5 d. **(D)** The Percentage Disease Index (PDI) was determined 3, 5, 7 and 10 days after infection (dai) of leaves as shown in panel C, left. The mock treatment was performed with sterile water. Bars represent means ± SEs, based on 4 x 24 leaves. Asterisks indicate significant differences as determined by the Student’s *t*-test (** P < 0.01). **(E) ***A. brassicae AbreATR1* transcript levels are higher in *cycam1-1* and *cycam1-2* than in WT leaves 5 dai. –*Ab*, unifected control, +*Ab*, *A. brassicae*-infected leaves. The plant *GAPDHC* gene served as control. The gel pictures are representative of 4 independent experiments with 3 replications each. **(F)** 14-day old WT, *cycam1-1* and *cam1-2* seedlings, which were either grown on MS medium (left) or MS medium supplemented with *A. brassicae* Tox preparation (Tox, right). The bottom pictures show Chl fluorescence images of the seedlings shown on the top. **(G)** Fresh weight of seedlings which were grown as demonstrated in panel **(F)**. In addition to the Tox, also the CWE, EPM or EPS preparations were tested. Data are means ± SEs from 5 independent experiments with >40 seedlings per treatment in each experiment (** P < 0.01).

Since the *cycam1* mutants were obtained by screening the EMS mutated pMAQ2 line with the *A. brassicae* CWE, we tested whether they are more susceptible to *A. brassicae* infections than WT. 14 d-old seedlings or leaves from 4 week-old plants were infected with *A. brassicae*. Roots were infected by exposing them to a 5 mm fungal plug (cf. Material and Methods, Figure 4A). The leaves of the seedlings and adult plants were infected with 5 μl of a spore suspension (Figure 4B-C). The disease progression in the leaves measured as percentage disease index was determined 3, 5, 7 and 10 days after infection (Figure 4D). The experiments demonstrated that *cycam1-1* and *cycam1-2* were more sensitive to *A. brassicae* infection than WT (Figure 4A-D). The higher transcript level of the *A. brassicae Atr1* marker gene in *cycam1* indicates that the mutant cannot efficiently restrict fungal growth (Figure 4E). Comparable results were obtained when the leaves were infected with the Tox preparation (Figure 4C). This can also be demonstrated by growing WT and *cycam1* seedlings on media containing low concentrations of the Tox preparation (Figure 4F). False colour images of the plates representing Fs/Fm values confirm that WT seedlings barely suffer under the applied Tox concentration while *cycam1-1 and cycam1-2* do (Figure 4F). Taken together, CYCAM1 is essential to establish resistance against *A. brassicae* infection and its Tox preparation. Since the CWE, EPM and EPS fractions, which induce [Ca^2+^]_cyt_ elevation, do not induce toxic effects on the plants or effect seedling’s growth, while the Tox preparation induces [Ca^2+^]_cyt_ elevation and toxicity (Figure 4G), their roles are different.

To test whether the lack of the Ca^2+^ response to exudate preparations from the pathogens *R. solani* and *P. parasitica* var. *nicotianae* has an influence on the resistance of Arabidopsis, 14 d-old *cycam1-1*, *cycam1-2* and WT seedlings were exposed to a fungal plug of these pathogens. The disease progression was significantly faster for the mutants compared to WT (Additional file 1: Figure S4). These data support the idea that *cycam1* is more susceptible to pathogens.

### *cycam1* is sensitive to ABA, salt and drought stress

**Figure 5:**
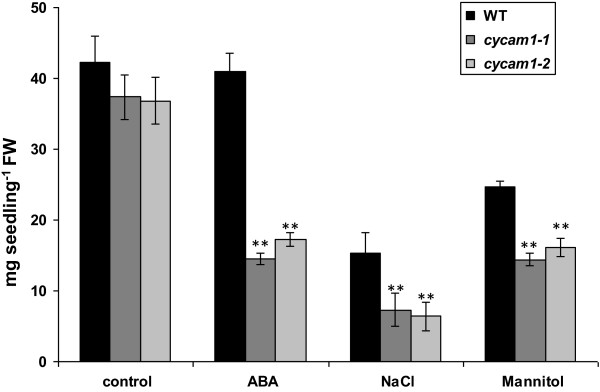
***cycam1 *seedlings are sensitive to abiotic (ABA, NaCl and mannitol) stress.** WT and *cycam1* seedlings were grown on MS medium supplemented with 100 nM ABA, 100 mM NaCl or 350 mM mannitol for 21 days before their fresh weights were determined. WT and *cycam1* seedlings grown on MS medium alone served as control. The values are means ± SEs of four independent experiments with > 40 seedlings of each line per experiment. Asterisks indicate significant differences as determined by the Student’s *t*-test (** P < 0.01).

When WT, *cycam1-1* and *cycam1-2* plants were grown on MS medium with 100 nM ABA, 100 mM NaCl or 350 mM mannitol for 3 weeks, their fresh weights were reduced compared to plants which were not exposed to stress. However, the extent of the reduction was much stronger for the mutant than for WT (Figure 5). The impaired fitness of the mutants can be demonstrated by measuring chlorophyll (Chl) fluorescence parameters which show that the efficiency of the photosynthetic electron flow is more impaired in stress-exposed mutants than in WT plants (Additional file 1: Figure S5). This indicates that *cycam1-1* and *cycam1-2* are more sensitive to ABA, salt and mannitol stress than WT.

### *cycam1* accumulates reactive oxygen species (ROS)

**Figure 6:**
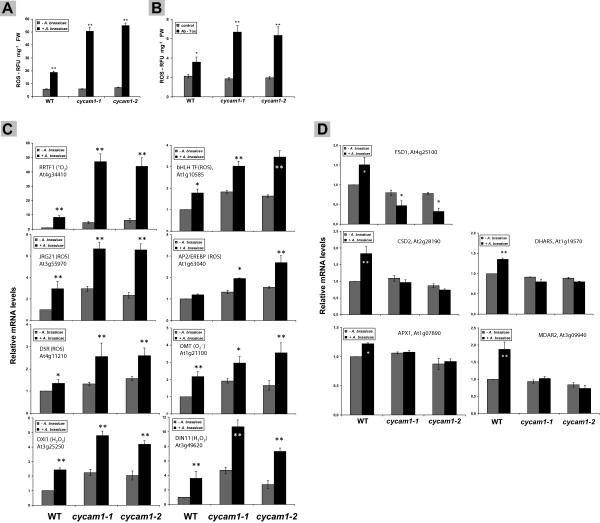
***cycam1-1 *and *cycam1-2 *accumulate more reactive oxygen species (ROS) than the WT after *A. brassicae *infection (A) or application of the *A. brassicae *Tox (B).** Roots of twelve-day old WT and *cycam1* seedlings were inoculated with 5 μl *A. brassicae* spore suspension containing 10^4^ to 10^5^ cfu ml^−1^**(A)** or with the *A. brassicae* Tox **(B)** and the ROS levels were measured in the roots 2 days later. Relative expression of ROS marker genes **(C)** or ROS scavenging genes **(D)** in WT and *cycam1* roots 2 days after infection with *A. brassicae* spores as described for **(A)**. The annotated functions of the genes/proteins are given in brackets [H_2_O_2_; singlet oxygen, (^1^O_2_); superoxide anion radical, ($O_{2}^{.-}$)]. For gene abbreviations, cf. TAIR (http://www.arabidopsis.org/). The mRNA levels of mock-treated WT seedlings were taken as 1.0 and the other values are expressed relative to it. All values are means ± SEs relative to the level of the root *GAPDHC* mRNA levels. Based on 3 independent experiments with 24 seedlings per experiment. Asterisks indicate significant differences, as determined by Student’s *t*-test (* P < 0.05; ** P < 0.01).

The amount of ROS in unchallenged *cyam1* roots is comparable to the amount in WT roots. However, after exposure to *A. brassicae* spores (Figure 6A) for 2 days or an *A. brassicae* Tox treatment (Figure 6B), the ROS level increases to significantly higher levels in the *cycam1* roots compared to the WT control. A stimulatory effect of the *A. brassicae* treatment was also observed for the expression of marker genes for different ROS species, although the pattern does not always match the pattern observed for the accumulation of the ROS species (Figure 6C). *A. brassicae* significantly stimulated the expression of *REDOX-REGULATED TRANSCRIPTION FACTOR1* (*RRTF1*), a marker gene for singlet oxygen accumulation, *OXIDATIVE SIGNAL INDUCIBLE1* (*OXI1*), a root specific marker gene for H_2_O_2_ accumulation, *JASMONATE-REGULATED GENE21* (*JRG21*), *DISEASE-RESISTANCE RESPONSIVE* (*DSR*) and *DARK-INDUCIBLE11* (*DIN11*), which represent general ROS marker genes (Figure 6C) [34-37]. A lower, but significant response was also observed for *INDOLE GLUCOSINOLATE O-METHYL TRANSFERAE1* (*OMT1*), a marker gene for $O_{2}^{.-}$. A comparative analysis of the mRNA data shown in Figure 6C demonstrates that some genes are already upregulated in unchallenged *cycam1* seedlings relative to the WT control and this effect is further promoted by the pathogen (e.g. *JRG21*, *OXI1*, *DIN11*), while in other cases it is primary the pathogen infection that stimulates the accumulation of the mRNAs in the mutant seedlings (most obvious for *RRTF1*). Apparently, the ROS-related genes respond differently to changes in the ROS levels, which might be due to the different regulation in response to the different ROS. Furthermore, the higher ROS levels after *A. brassicae* infection may be partially caused by less efficient ROS scavenging, since the mRNA levels for several ROS scavenging enzymes which are upregulated in WT roots after *A. brassicae* infection, are not upregulated in the roots of the *cycam1* mutant (Figure 6D).

### Phytohormone levels are altered in *cycam1*

**Figure 7:**
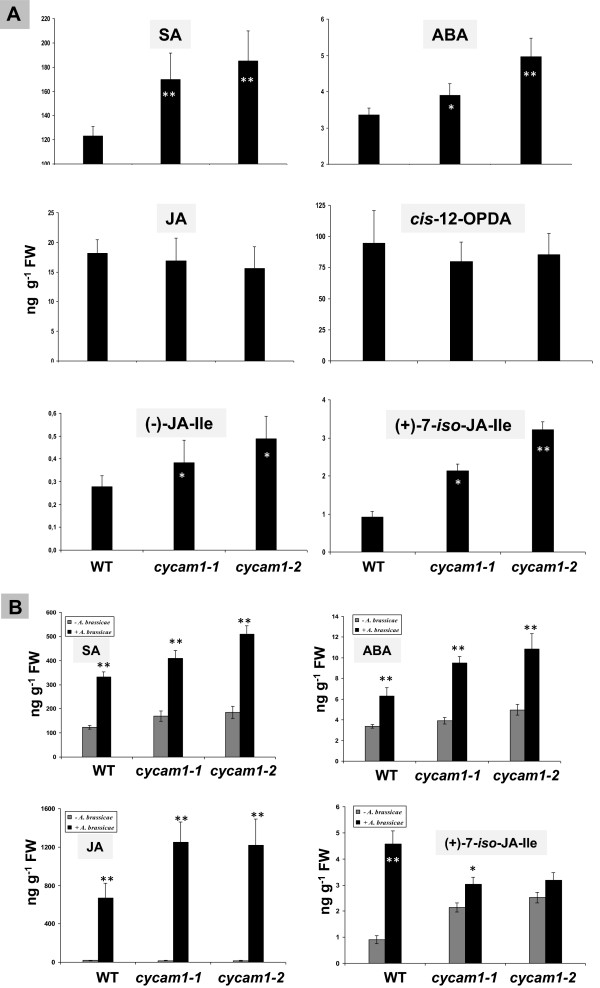
**Phytohormone levels in WT and *cycam1 *seedlings.** WT, *cycam1-1* and *cycam1-2* seedlings were grown on MS medium under LD condition for 14 days. SA, ABA, JA, *cis*-OPDA, (-)-JA-Ile and (+)-7-iso-JA-Ile levels were determined in total seedlings. The values are means ± SEs of four independent experiments with five replications in each experiment **(A)**. The leaves of 12-day old WT, *cycam1-1* and *cycam1-2* seedlings grown on MS under LD conditions were inoculated with a fungal spore suspension containing 10^4^ to 10^5^ cfu ml^−1^ and incubated under LD conditions for 3 additional days. SA, ABA, JA and (+)-7-iso-JA-Ile levels were determined in total seedlings. The values are means ± SEs of four independent experiments with five replications in each experiment **(B)**. Asterisks indicate significant differences, as determined by Student’s *t*-test (* P < 0.05; ** P < 0.01).

The phytohormones salicyclic acid (SA), jasmonic acid (JA) and ABA play crucial roles in regulating growth and development and coordinate the plant’s responses to biotic and abiotic stresses [38-40]. SA-, JA- and ABA-dependent stress responses are regulated by [Ca^2+^]_cyt_ levels in plants [15,41-46]. To check whether the SA, JA and ABA levels are altered in the mutant, their levels were first measured in 14 d-old *cycam1-1, cycam1-2* and WT seedlings grown on MS medium. The SA and ABA levels were slightly, but significantly higher in *cycam1-1* and *cycam1-2* seedlings not exposed to stress compared to the WT control (Figure 7A). The JA level and that of its precursor *cis*-12-oxo-phytodienoic acid (*cis*-12-OPDA) were not affected by the mutation (Figure 7A). However, the inactive form jasmonoyl-isoleucine (JA-Ile) conjugate, (-)-JA-Ile [47], and the bioactive form (+)-7-iso-JA-Ile [48] were higher in *cycam1-1* and *cycam1-2* compared to the WT (Figure 7A). This suggests that JA-modifying enzymes, but not JA synthesis, are targets of the *cycam1* mutation. In conclusion, the levels of SA, ABA and the bioactive (+)-7-iso-JA-Ile are higher in the Ca^2+^ mutants, even when they are not exposed to stress.

*A. brassicae* infection induced SA, ABA and JA accumulation in WT and *cycam1* seedlings (Figure 7B). Induction of the phytohormone levels is quite similar in WT and the *cycam1* mutant, when the %-stimulation by the pathogen is considered, except that the biologically active form of JA, (+)-7-*iso*-JA-Ile, is induced more strongly in infected WT than *cycam1* seedlings (Figure 7B). The levels of SA, ABA and JA are almost identical in WT and mutant seedlings, while those of (+)-7-*iso*-JA-Ile are twice as high in the mutant compared to the WT control (Figure 7B). The SA-inducible *NPR1* and *PR1* (Additional file 1: Figure S6A), the ABA-inducible *BG1*, *NCED3* and *TOC1* (Additional file 1: Figure S6B) and the JA-inducible *JAZ1* were not or not significantly higher expressed in the unchallenged allelic mutants compared to the unchallenged WT control, whereas a minor stimulation could be observed for the JA-inducible *MYC2*, *VSP2*, *Thi2*, *PDF1.2* and *JASMONATE_REGULATED GENE21* (*JRG21*, Additional file 1: Figure S6C). Furthermore, in almost all cases, the % induction of these mRNA levels by *A. brassicae*, three days after infection of the leaves with the spores, is comparable for WT and mutant seedlings. Therefore, it appears that the higher mRNA levels are mainly caused by the pathogen and not by the mutation. No significant differences could be detected for the *ABA1* and *ABA2* mRNA levels.

**Figure 8:**
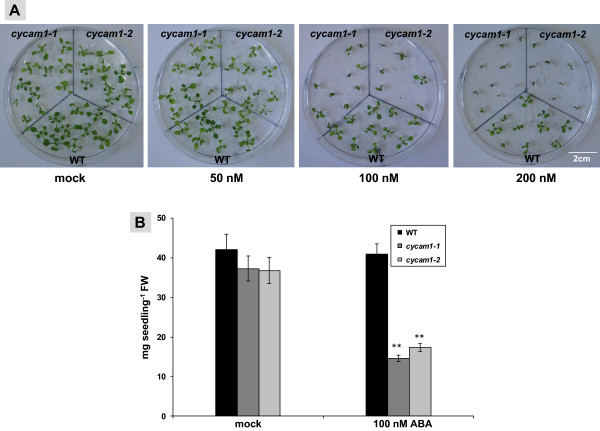
**ABA inhibits germination and growth of *cycam1 *seedlings.** WT, *cycam1-1* and *cycam1-2* seedlings were grown on MS medium supplemented with 50, 100 and 200 nM ABA under LD conditions. ABA (100-200 nM) inhibited germination and growth of *cycam1-1* and *cycam1-2* more than the WT **(A)**. At 200 nM ABA, the expansion of *cycam1*, but not WT cotyledons was inhibited **(A)**. Fresh weight of WT, *cyam1-1* and *cycam1-2* seedlings exposed to 100 nM ABA (or mock treatment) for 3 weeks. **(B)**. Asterisks indicate significant differences, as determined by Student’s *t*-test (** P < 0.01).

The elevated phytohormone levels in unstressed *cycam1-1* and *cycam1-2* prompted us to investigate the response of the Ca^2+^ mutant to exogenous application of SA, methyl jasmonate (MeJA) and ABA. The phytohomones were added to the MS medium in optimized concentrations. Application of SA or MeJA (5 and 100 μM, respectively) did not cause any difference in the growth of WT and *cycam1* seedlings. However, ABA (100-200 nM) inhibited germination and growth of *cycam1-1* and *cycam1-2* more than WT. At 200 nM ABA, the expansion of *cycam1*, but not WT cotyledons was strongly inhibited (Figure 8A). Three weeks after treatment with 100 nm ABA, the biomass of *cycam1* seedlings was less than half of the biomasses of WT seedlings (Figure 8B). Thus, the elevated ABA level already present in the mutants in addition to the exogenous application of ABA is deleterious for the mutants. It is interesting to note that also in the presence of ABA, no Ca^2+^ response was observed in the *cycam1* mutant in response to the fungal stimuli.

### *A. brassicae* affects camalexin and glucosinolate levels

**Figure 9:**
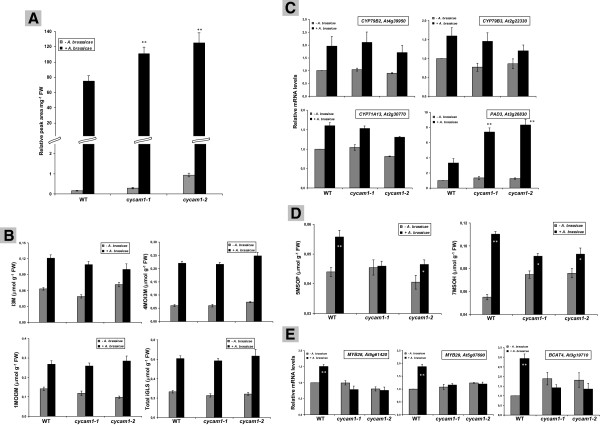
**Induction of camalexin, indolic glucosinolates (iGLS), aliphatic glucosinolates (aGLS) and the genes involved in their biosynthesis in WT and *cycam1 *after *A. brassicae *infection.** The leaves of 12-day old WT, *cycam1-1* and *cycam1-2* seedlings grown on MS under LD conditions were transferred to PNM media as described in Methods, inoculated with a fungal spore suspension containing 10^4^ to 10^5^ cfu ml^−1^ and incubated under LD condition for additional 3 days before determination of camalexin (relative quantification) **(A)**, iGLS **(B)** and aGLS **(D)** levels. iGLS are I3M (indolyl-3-methyl-GLS), 4MOI3M (4-methoxyindolyl-3-methyl-GLS) and 1MOI3M (1-methoxyindolyl-3-methyl-GLS). aGLS are 5MSOP (5-methylsulfinylpentyl-GLS) and 7MSOH (7-methylsulfinyl-heptyl-GLS). mRNA levels of camalexin and iGLS **(C)** and aGLS **(E)** biosynthesis genes in the leaves of WT, *cycam1-*1 and *cycam1-2* 2 dai with *A. brassicae*. Mock treatment was performed with sterile water. The abbreviations of the gene and annotation numbers are given. The mRNA levels for each cDNA were normalized with respect to the plant *GAPDHC* message levels. The mRNA level for mock-treated WT seedlings was set as 1.0 and the other values refered to it. The values are means ± SEs of four independent RT-PCR experiments with three replications in each experiment. For **(A)**, **(B)** and **(C)**, all values (+ *A. brassicae*) are significantly different from the values (- *A. brassicae*) with P < 0.01. For **(D)** and **(E)**, asterisks indicate significant differences, as determined by Student’s *t*-test (* P > 0.1; ** P < 0.01).

Camalexin and glucosinolates are major sulphur containing secondary metabolites involved in plant defense in Arabidopsis [49,50]. *A. brassicae* infection induced both camalexin and indolic glucosinolates (iGLS) and their biosynthesis genes in the WT and mutant (Figure 9A-C). The induction of the aliphatic glucosinolates (aGLS) 3-methylthiobutyl-GLS, 4-methyl sulfinylbutyl-GLS, 4-methylthiobutyl-GLS and 8-methylsulfinyl-octyl-GLS (data not shown) was not significantly different between WT and mutant seedlings, while the aGLS 5-methylsulfinylpentyl-GLS (5MSOP) and 7-methylsulfinylheptyl-GLS (7MSOH) levels were higher in the WT than the mutants (Figure 9D). The expression of *MYB28*, *MYB29* and *BCAT4* which are involved in aGLS biosynthesis [49] were also upregulated in the WT and not in the mutant after *A. brassicae* infection (Figure 9E). This shows that aGLS biosynthesis is less efficiently induced in *cycam1*.

## Discussion

Exudate preparations from *A. brassicae*, *R. solani*, *P. parasitica*, and *A. tumefaciens* induce [Ca^2+^]_cyt_ elevation in Arabidopsis roots as monitored with the bioluminescent Ca^2+^ binding protein aequorin (Figure 1-3, Additional file 1: Figures S1 and S3). Characterization of the Ca^2+^ signatures induced by these stimuli demonstrates that they resemble those described for many MAMPs from various plant species: β-glucan from *P. sojae* in soybean cell cultures [51], pep-13 from *Phytophthora sojae* in parsley cell cultures [12], harpin from *Pseudomonas syringae* pv. *phaseolina* in tobacco [52], a yeast elicitor and chitosan in Arabidospsis [18], cryptogein from *P. cryptogea* and oligosaccharides in tobacco cell cultures [2,14], pep-25 from *P. sojae* in Arabidopsis seedlings [15], INF1 from *P. infestans* and boehmerin from *P. boehmeriae* in tobacco [53], flg22 from flagellated bacteria and elf18 from the elongation factor Tu in Arabidopsis seedlings [54,55].

Here we describe an *A. thaliana* mutant which fails to induce [Ca^2+^]_cyt_ elevation in Arabidopsis roots in response to the exudate preparations from pathogenic root-interacting microbes. The chemical components which induce [Ca^2+^]_cyt_ elevation are either present in cell wall preparations from these microbes or released into the medium from mycelia or germinating spores. Although these chemical mediators have not yet been determined, the shape of their Ca^2+^ signatures, their dose-dependency and refractory nature demonstrate that they require CYCAM1 for function (Figure 3). The *cycam1* mutant is not impaired in the response to flg22 and to a CWE from the root-colonizing fungus *M. hyalina*, indicating some specificity of Arabidopsis response to pathogen exudates. Like flg22 and the Myc factor [31,56,57], the active components in the *A. brassicae* exudate preparations are thermostable, hydrophilic, polar and of low molecular weight (Additional file 1: Figure S2).

Interestingly, the CWEs, EPM and EPS preparations from *A. brassicae* induce [Ca^2+^]_cyt_ elevation (Figure 1), but not the typical disease symptoms of the fungus in Arabidopsis, while the Tox preparation from *A. brassicae* induces [Ca^2+^]_cyt_ elevation (Figure 1) and is toxic (Figure 4C, F). Toxs from pathogenic fungi including *A. brassicae* are known to disrupt membranes [21,22] which might also contribute to the Ca^2+^ influx into the cytoplasm. This might also explain the slower recovery of the Ca^2+^ signal after Tox application than after application of CWE, EPM and EPS preparations. The Ca^2+^ response induced by the non-toxic CWE, EPM or EPS might establish a first line of defense that is then followed by a second stronger response induced by the Tox.

CYCAM1 also plays a role in abiotic stress as demonstrated by the increased sensitivity of *cycam1* seedlings to ABA, salt and mannitol applications (Figure 5). [Ca^2+^]_cyt_ elevation is well documented in response to drought stress [18,58,59]. Both ABA and H_2_O_2_ induce [Ca^2+^]_cyt_ elevations in guard cells to regulate stomata aperture [1,18,60,61]. Sustained [Ca^2+^]_cyt_ elevations induced by mannitol are required for tolerance to drought and osmotic stress in Arabidopsis [58,59]. Therefore, CYCAM1 is involved in both biotic and abiotic stress responses. It appears that the higher stress sensitivity of *cycam1* is associated with imbalances in redox and ROS homeostasis since the mutant accumulates more ROS after *A. brassicae* infection than the WT (Figure 6A). Since this response can also be induced by the Tox (Figure 6B), the pathogen is not required. Several ROS marker genes representative for different ROS species are more strongly upregulated in the *A. brassicae*-exposed mutant than in the WT (Figure 6C) which is consistent with the idea that a general stress response cannot be efficiently repressed in the mutant. A quite strong stimulatory effect by *A. brassicae* in the mutant is observed for *RRTF1*, a marker gene for singlet oxygen accumulation, while *OXI1*, which codes for a root-specific kinase induced in response to H_2_O_2_ treatment and H_2_O_2_-generating stimuli, *JRG21*, a general ROS marker, the bHLH transcription factor gene *At1g10585* and *DIN11* are already higher in the unchallenged mutant compared to the WT control and further upregulated in *A. brassicae*-exposed WT and mutant seedlings (Figure 6C) [62]. Since the % induction is comparable in WT and mutant seedlings, the expression is promoted by the mutation and this effect is further stimulated after pathogen infection. The higher ROS accumulation is partially caused by the inability of the mutant to efficiently scavenge the accumulation of ROS, several genes for ROS scavenging enzymes which are upregulated in WT roots, are not upregulated in the mutant roots (Figure 6D).

To initially characterize the role of CYCAM1, we measured the ABA, SA and JA levels in untreated mutant seedlings and those exposed to *A. brassicae* infections or to the Tox preparations. These three hormones play key roles in mediating disease responses to necrotrophic and biotrophic pathogens. *cycam1* accumulates higher ABA, SA and bioactive JA derivative levels compared to WT (Figure 7A). Interaction studies with biotrophic, hemibiotrophic and necrotrophic pathogens on ABA-deficient mutants demonstrate that ABA is a negative regulator of plant defense [42,63-65]. The hypersusceptibility of *cycam1* to *A. brassicae*, its Tox and the other microbes tested confirms a link between CYCAM1-mediated [Ca^2+^]_cyt_ elevation, ABA and innate immunity. The ABA level was higher in the two allelic *cycam1* mutants when they were not exposed to stress (Figure 7A), and these mutants become even more sensitive to exogenously applied ABA compared to WT (Figure 8A). The ABA biosynthesis genes *BG1*, *NCED3* and *TOC1* were higher in *A. brassicae*-exposed *cycam1* mutants than in the WT, whereas the *ABA1* and *ABA2* mRNA levels did not show a significant difference (Additional file 1: Figure S6B). BG1, a β-glucosidase located in the endoplasmic reticulum, hydrolyzes glucose conjugated, biologically inactive ABA to produce active ABA [66]. NCED3, a 9-*ci*s-epoxycarotenoid dioxygenase and TIMING OF CAB EXPRESSION1 (TOC1) are involved in *de novo* ABA synthesis [64,67]. Therefore, elevated ABA levels in *A. brassicae*-exposed *cycam1* mutants may be caused by a higher *de novo* synthesis and the conversion of inactive ABA to its active form. Exposure of *cycam1* with elevated ABA levels to even more exogenously applied ABA leads to more severe lesions, as shown by the germination and growth assays on ABA-containing media (Figure 8).

*A. brassicae* infection induced SA (Figure 7B) and SA-responsive gene *PRI* (Additional file 1: Figure S6A) in *cycam1* and WT seedlings. SA has both negative and positive roles in plant defense against fungal and bacterial pathogens [40,68] and references therein]. The *phospholipase Dβ1 (pldβ1)* mutant and mutants impaired in phosphatidic acid (PA) biosynthesis were more susceptible to *B. cinerea* infection compared to the WT and this was associated with a higher SA level in the infected mutant plants [69], similar to our observations with *cycam1*. PLDβ1 binds Ca^2+^, hydrolyzes phospholipids to generate PA and is involved in hormone signaling [53] and the response to disease resistance [69-71]. Therefore, the slightly elevated SA levels in unchallenged *cycam1* suggest that [Ca^2+^]_cyt_ elevation restricts SA accumulation, which becomes harmful if the mutant is exposed to SA-stimulating biotic and abiotic stress.

JA, methyl-JA and other bioactive derivatives are important molecules in regulating induced defense responses against necrotrophic pathogen infection [38,72]. *A. brassicae* infection induced higher JA levels in *cycam1* than in WT seedlings, while the levels in unchallenged WT and mutant seedlings is almost identical (Figure 7B). Therefore, JA may act as a positive regulator of enhanced susceptibility to *A. brassicae* in *cycam1*. The role of JA in disease susceptibility to *A. alternata* f. sp. *lycopersici* (AAL) and its AAL-Tox is well established for tomato [73]. Furthermore, JA promoted AAL-Tox-induced cell death through JA INSENSITIVE1 (JAI1) receptor-dependent JA signalling [74]. The expression of the JA-responsive genes *MYC2*, *VSP2*, *JAZ1*, *Thi2.1* and *PDF1.2* was slightly higher in *A. brassicae* infected *cycam* than WT seedlings (Additional file 1: Figure S6C). The higher mRNA levels for the marker genes of the MYC (*VSP2*) and ERF (*PDF1.2*) branch of the JA pathway in *cycam1* suggests that both branches are regulated by CYCAM1. In addition, the expression of the *JRG21*, a common ROS marker gene involved in biotic and abiotic stress and JA signaling [36,37], was higher in unchallenged *cycam1* and WT seedlings, and the presence of *A. brassicae* led to a similar %-age increase in the mRNA levels for both WT and mutant seedlings (Additional file 1: Figure S6C). These findings suggest that CYCAM1 is involved in control of JA accumulation and signaling. Furthermore, the aGLS biosynthetic genes *BCAT4* (*BRANCHED-CHAIN AMINOACID AMINO TRANSFERASE4*), *MYB28* and *MYB29*[75] were higher in *A. brassicae*-infected WT seedlings compared to *A. brassicae*-infected *cycam1* seedlings (Figure 9E). This suggests that the aGLS-synthesizing genes play an important role in defense against *A. brassicae* infection in Arabidopsis mediated through *CYCAM1*.

## Conclusions

**Figure 10:**
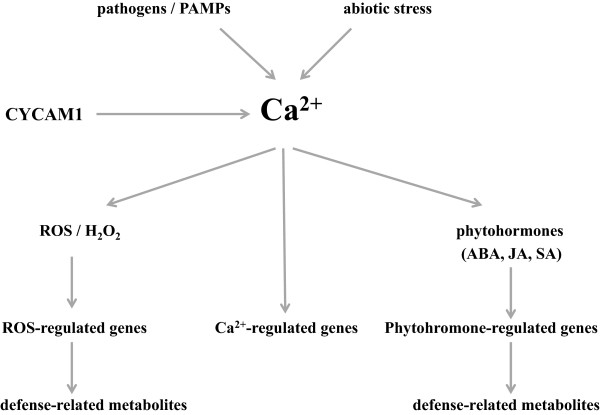
A scheme describing the events which are affected by CYCAM1.

We isolated a mutant which does not induce [Ca^2+^]_cyt_ elevation in response to different pathogenic fungal exudates. CYCAM1 is involved in [Ca^2+^]_cyt_-mediated abiotic and biotic stress responses (Figure 10). The *cycam1* mutant accumulates higher levels of the biologically active phytohromones SA, ABA and (+)-7-*iso*-JA-Ile, is sensitive to exogenous ABA applications and accumulates more ROS than WT after *A. brassicae* infection, although the ROS levels in the unchallenged WT and mutant seedlings are comparable. The Ca^2+^ response in the WT can be induced by the non-toxic CWE, EPM or EPS which might establish a first line of defense, followed by a stronger defense response induced by the Tox.

## Methods

### Plant material and growth

Transgenic *Arabidopsis thaliana* expressing cytosolic apoaequorin (Aeq^cyt^) in Col-0 background (pMAQ2) was a gift from Prof. Marc Knight [32]. Mutagenesis was performed using 0.2% ethyl-methane sulfonate (w/v). Individual M_2_ seeds were grown on Hoagland (HL) medium containing 1% agar in square plates (120 × 120 × 16 mm; Nerbe Plus GmbH Germany). After stratification at 4°C for 48 h, plates were kept vertically to grow the roots on the surface of the medium and incubated for 18 days under long day (LD) conditions (16h/8h, light/dark; temperature, 20 ± 1°C; light intensity, 80 μmol m^−2^ sec^−1^) [32,76].

### Mutant screen and [Ca^2+^]_cyt_ measurement

Aequorin based luminescence measurements were performed using 16-day old individual M_2_ plants grown in Hoagland medium [32,76]. pMAQ2 plants served as control [30]. For [Ca^2+^]_cyt_ measurements, approximately 70% of the roots per seedling were dissected and incubated overnight in 150 μl of 7.5 μM coelenterazine (native CTZ, P.J.K. GmbH, Germany, No 102171) in the dark at 20°C in a 96 well plate (Thermo Fischer Scientific, Finland, Cat. no. 9502887). For cotyledon assays, the same protocol was used except that the root material was replaced by 3 leaves of the seedlings grown under the same conditions. For the leaf assay, ~ 1/32 part of a fully developed leaf (without middle rib) of 4 week-old plants grown in pots under LD conditions were used. Bioluminescence counts from roots or cotyledons/leaves were recorded as relative light units (RLU) with a microplate luminometer (Luminoskan Ascent, version 2.4, Thermo Electro Corporation, Finland). The mutant screen was performed with the CWE from *A. brassicae*; the putative M_2_ mutants were rescued and transferred to pots containing garden soil and vermiculite at 9:1 (v/v) for further screening and validation. The mutant seedlings were grown in a temperature-controlled growth chamber under short day (SD) condition (8h/16h, light/dark; temperature, 20 ± 1°C; light intensity, 80 μmol m^−2^ sec^−1^) for 4 weeks followed by LD condition in Aracon tubes. The seeds were harvested from individual M_3_ plants and again screened to obtain homozygote mutants.

### Growth and maintenance of fungi

*A. brassicae* (FSU-3951) was obtained from Jena Microbial Resource Centre, Jena, Germany. The fungus was grown on potato dextrose agar (PDA) medium (pH 6.5-6.7) at 20 ± 1°C in a temperature-controlled chamber under 12/12 h light/dark and 75% relative humidity for 2 weeks. To maintain the virulence, the fungus was inoculated to Arabidopsis seedlings and re-isolated from the infected tissues periodically [77].

### Preparation of *A. brassicae* spore suspension

*A. brassicae* sporulates heavily in Potato Dextrose Broth (PDB; pH 6.5-6.7). A two-week-old fungal plug (5 mm diameter) was inoculated to PDB and incubated for 2 weeks. The medium was removed by filtering through 4 layers of sterilized nylon membrane and the hyphae and spores were washed 3 times with sterile H_2_O to remove the residual medium. The spores and hyphae were gently homogenized with 50 ml of sterile H_2_O and filtered through four layers of sterilized nylon membrane. The spore concentration was adjusted to 10^4^-10^5^ colony forming units (cfu) ml^−1^ by serial dilutions and counting with a Haemocytometer. For uniform dispersion of spores, 1-2 drops of Tween-20 was added to 100 ml of spore suspension.

### Inoculation of *A. brassicae* to roots, cotyledons and mature leaves

For root infection, 12-day old seedlings were transferred to fresh PNM plates with a sterilized nylon membrane [78]. A five mm fungal plug from 2-week-old *A. brassicae* was kept 1 cm away from the roots. The plates were sealed with Parafilm and incubated in a temperature controlled growth chamber under LD condition. Leaf infections were performed 48 h after the transfer of 12-d old seedlings to PNM plates. Six leaves in the middle whorl of the seedlings were inoculated with 5 μl of spore suspension containing 10^4^-10^5^ cfu ml^−1^. Infection of mature leaves was performed with detached leaves. They were detached from 4 week-old plants grown under SD condition at 20°C and 80 μmol m^−2^ sec^−1^. Sterile Whatmann filter paper was placed on a Petri dish and 1 ml of sterile H_2_O was added to soak the filter paper. Five detached leaves were kept on the soaked filter paper and inoculated with 10 μl of the spore suspension containing 10^4^ to 10^5^ cfu ml^−1^ directly on to leaves. Mock treatment was performed with sterile H_2_O. The plates were sealed with Parafilm and incubated under LD conditions as described above. The progression of disease development was determined as Percentage Disease Index (PDI) at 3, 5, 7 and 10 days after infection using standard disease intensity grades. For the Tox treatment, 10 μl of the *A. brassicae* Tox preparation was applied directly on the detached leaves; mock treatment was performed with sterile H_2_O.

### Preparation of CWE from *A. brassicae* and *A. tumefaciens*

The CWE was prepared according to Anderson-Prouty and Albersheim [79] with modifications [32,76]. Mycelia from liquid cultures were harvested by filtration through 4 layers of nylon membrane (pore size, 70 μm; mesh count, 92 cm^−1^; Sefar GmbH, Switzerland) and washed 5 times with sterile H_2_O. The mycelia were homogenised in sterile H_2_O (1:5; w/v) with a Waring blender, and the homogenate was filtered through four layers of nylon membrane. The residue was collected and again washed three more times with sterile H_2_O; twice with chloroform/methanol (1:1) and finally twice with acetone. The mycelial cell wall (CW) was air-dried for 2 h under sterile conditions. The CWE was prepared from the dried mycelial CW by suspending 1 g of CW material in 100 ml sterile H_2_O and autoclaving for 30 min. After cooling, the extract was filtered through 4 layers of nylon membrane, then through 2 layers of Whatman filter paper and finally filter-sterilised using a 0.22 μm filter to remove undissolved substances. The fungal CWE was further purified by passing it through a reversed phase Supelclean LC-18 SPE cartridge (10 g bed weight; 60 ml volume; 60 A pore size; Sigma-Aldrich, Taufkirchen, Germany, Cat. No. 57136). The fractions were identified by [Ca^2+^]_cyt_ elevation measurements and combined [76,80]. Two-day old spores of *A. tumefaciens* grown on yeast extract broth were harvested by centrifugation for preparing their CWE.

### Preparation of water diffusible exudate preparations from mycelia (EPM) and germinating spores (EPS)

*A. brassicae* mycelium, propagated on PD broth for 14 days, was filtered through four layers of sterilised nylon membrane and intensively washed seven times with sterile H_2_O to remove the medium and spores. After air drying of the mycelium for 1 h, it was re-suspended in sterile H_2_O and incubated at 20°C in a horizontally rotating shaker with 60 rpm. After 48 h, the mycelium was removed from the water by filtering through 4 layers of sterile nylon membrane, then through 2 layers of filter paper and finally filter-sterilized using a 0.22 micron pore size filter. The crude water-diffusible fraction was further purified by passing it through a Reverse Phase Supelclean LC-18 Cartidges to obtain the active and partially pure fractions as described above [76,80]. For the preparation of a water diffusible exudate fraction from germinating spores, 10^7^-10^8^ cfu ml^−1^ of the fungus were incubated in distilled water for 48 h at 20°C. During shaking with 60 rpm, more than 90% of the spores germinated. They were filtered through 4 layers of sterile nylon membrane, then 2 layers of filter paper and filter-sterilized using a 0.22 micron pore size filter. The filtrate was finally purified by passing it through a Reversed Phase Supelclean LC-18 cartidge.

### *A. brassicae* Tox preparation

A Tox fraction from *A. brassicae* culture filtrate was generated as described by Vidhyasekaran et al. [81] with modifications. Erlenmeyer flasks (250 ml) with 100 ml of PDB were inoculated with a 5-mm disc of mycelium grown on PDA plates and incubated at 22°C, relative humidity 75%, and 12h/12h light/dark cycle with a light intensity of 80 μmol m^−2^ sec^−1^. After 4-5 weeks, the culture filtrates were collected by filtering through 8 layers of sterile nylon membrane and twice through 2 layers of Whatman filter paper. The culture filtrate was concentrated to 1/10^th^ volume in vacuum at 40°C using a Rotavapor (Büchi Laboratoriums-Technik AG, Flawil, Switzerland). An equal volume of methanol (HPLC grade) was added and mixed well, and the solution was stored overnight at 4°C. Precipitates were removed by filtration through 4 layers of nylon membrane and then through Whatman filter paper. The aqueous fraction was extracted three times with equal volumes of trichloromethane, ethyl acetate, n-hexane and petroleum ether using a separation funnel. After filtration through 4 layers of nylon membrane, the aqueous fraction was centrifuged at 10,000 rpm for 10 min and the supernatant was filter-sterilized using a 0.22 μM filter. The Tox preparation was further purified by passing it through a Sephadex G100 column and the active fractions were collected and lyophilised. The powder was re-suspended in sterile H_2_O and further purified by passing it through a reversed phase Supelclean LC-18 SPE cartridge. The active fractions were collected and used as stimulus for [Ca^2+^]_cyt_ measurements and physiological studies.

### Germination, growth of seedling and root assays

The surface-sterilized seeds of WT (pMAQ2) and the *cycam1* mutant were placed on MS medium [82]. For drought stress experiments, different concentrations of mannitol or NaCl were added before autoclaving. Different concentrations of ABA were added after autoclaving. As control, WT and *cycam1* mutants were grown on MS medium alone. After cold treatment at 4°C for 48 h, plates were incubated at 20°C under LD condition and 80 μmol m^−2^ sec^−1^, as described in the text. For root assays, different concentrations of filter-sterilized methyl-JA and SA solutions were added to sterilized HL medium to obtain the required final concentrations and seeds were plated on it [45].

### Measurement of photosynthesis parameter

False color pictures of Chl fluorescence images representing Fs/Fm values of seedlings in plates were obtained as described by Wagner et al. [83]. Blue represents low F_s_/F_m_ values above a threshold of 0.06 and red represents high F_s_/F_m_ values with an upper threshold limit of 0.17.

### Phytohormone measurement

100 mg of leaf material was frozen in liquid nitrogen and kept at -80˚C. After grinding with mortar and pestle, the leaf material was extracted with 1.2 ml of methanol containing 24 ng of 9,10-D_2_-9,10-dihydrojasmonic acid, 24 ng D_4_-salicylic acid (Sigma-Aldrich), 24 ng D_6_-abscisic acid (Santa Cruz Biotechnology, Santa Cruz, U.S.A.), and 4.8 ng of JA-^13^C_6_-Ile conjugate as internal standards. JA-^13^C_6_-Ile conjugate was synthesized as described by Kramell et al. [84] using ^13^C_6_-Ile (Sigma-Aldrich). The homogenate was mixed for 30 min and centrifuged at 14,000 rpm for 20 min at 4°C. The supernatant was collected. The homogenate was re-extracted with 500 μl methanol, mixed well, centrifuged and supernatants were pooled. The combined extracts was evaporated in a speed-vac at 30°C and re-dissolved in 250 μl methanol. Chromatography was performed on an Agilent 1200 HPLC system (Agilent Technologies). Separation was achieved on a Zorbax Eclipse XDB-C18 column (50 × 4.6 mm, 1.8 μm, Agilent). Formic acid (0.05%) in water and acetonitrile were employed as mobile phases A and B, respectively. The elution profile was: 0-0.5 min, 5% B; 0.5-9.5 min, 5-42% B; 9.5-9.51 min 42-100% B; 9.51-12 min 100% B and 12.1-15 min 5% B. The mobile phase flow rate was 1.1 ml/min. The column temperature was maintained at 25°C. An API 3200 tandem mass spectrometer (Applied Biosystems) equipped with a Turbospray ion source was operated in negative ionization mode. The instrument parameters were optimized by infusion experiments with pure standards, where available. The ionspray voltage was maintained at -4500 eV. The turbo gas temperature was set at 700°C. Nebulizing gas was set at 60 psi, curtain gas at 25 psi, heating gas at 60 psi and collision gas at 7 psi. Multiple reaction monitoring (MRM) was used to monitor analyte parent ion → product ion: m/z 136.9 → 93.0 [collision energy (CE) - 22 V; declustering potential (DP) - 35 V] for SA; m/z 140.9 → 97.0 (CE - 22 V; DP - 35 V) for D4-SA; m/z 209.1 → 59.0 (CE - 24 V; DP - 35 V) for JA; m/z 213.1 → 56.0 (CE - 24 V; DP - 35 V) for 9,10-D2-9,10-dihydrojasmonic acid; m/z 263.0 → 153.2 (CE - 22 V; DP - 35 V) for ABA; m/z 269.0 → 159.2 (CE - 22 V; DP - 35 V) for D6-ABA; m/z 322.2 → 130.1 (CE - 30V; DP - 50V) for JA-Ile conjugate; m/z 328.2 → 136.1 (CE - 30V; DP - 50V) for JA-^13^C_6_-Ile conjugate. Both Q1 and Q3 quadrupoles were maintained at unit resolution. Analyst 1.5 software (Applied Biosystems) was used for data acquisition and processing. Linearity in ionization efficiencies were verified by analyzing dilution series of standard mixtures. Phytohormones were quantified relative to the signal of their corresponding internal standard. For quantification of 12-oxophytodienoic acid, *cis*-OPDA, 9,10-D_2_-9,10-dihydro-JA was used as the internal standard applying an experimentally determined response factor of 1.

### Quantification of camalexin by LC-MS

Samples were freeze-dried until constant weight and ground to a fine powder. Ten to fifty mg of freeze-dried and pulverised material was used for camalexin measurement by LC-MS analysis. Camalexin was analysed in the flow-through samples resulting from the extraction procedure for glucosinolate analysis (see below). In glucosinolate extraction, the raw extract was loaded onto DEAE Sephadex, and the resulting flow-through was collected in a 96 deepwell plate and directly analysed by LC-MS/MS. Chromatography was performed on an Agilent 1200 HPLC system (Agilent Technologies, Böblingen, Germany). Separation was achieved on a Zorbax Eclipse XDB-C18 column (50 × 4.6 mm, 1.8 μm, Agilent, Germany). Formic acid (0.05%) in water and acetonitrile were employed as mobile phases A and B, respectively. The elution profile was: 0-0.5 min, 5% B; 0.5-1 min, 5-100% B in A; 1-2 min 100% B and 2.1-4. 5 min 5% B. The mobile phase flow rate was 0.8 ml/min. The column temperature was maintained at 25°C. An API 3200 tandem mass spectrometer (Applied Biosystems, Darmstadt, Germany) equipped with a Turbospray ion source was operated in positive ionization mode. The instrument parameters were optimized by infusion experiments. The ionspray voltage was maintained at 5500 V. The turbo gas temperature was set at 700°C. Nebulizing gas was set at 70 psi, curtain gas at 35 psi, heating gas at 70 psi and collision gas at 2 psi. Multiple reaction monitoring (MRM) was used to monitor analyte parent ion → product ion: *m/z* 201.09 → 59.01 [collision energy (CE) 45 V; declustering potential (DP) 51 V]. Both Q1 and Q3 quadrupoles were maintained at unit resolution. Analyst 1.5 software (Applied Biosystems, Darmstadt, Germany) was used for data acquisition and processing. Linearity in ionization efficiencies was verified by analyzing dilution series of samples containing camalexin. A relative quantification of camalexin was performed by calculating peak area per mg of fresh weight.

### Determination of glucosinolates (GLS)

Samples were freeze-dried until constant weight and ground to a fine powder. Ten to fifty mg of freeze-dried and pulverised material per plant was used for GLS analysis. GLS were extracted with 1 ml of 80% methanol solution containing 0.05 mM intact 4-hydroxybenzyl GLS as internal standard and desulfated with arylsulfatase (Sigma-Aldrich) on a DEAE Sephadex A 25 column. The eluted desulfoglucosinolates were separated using high performance liquid chromatography (Agilent 1100 HPLC system, Agilent Technologies, Waldbronn, Germany) on a reversed phase column (Nucleodur Sphinx RP, 250 × 4.6 mm, Macheray-Nagel, Düren, Germany) with an water-acetonitrile gradient (1.5% acetonitrile for 1 min, 1.5-5% acetonitrile from 1-6 min, 5-7% acetonitrile from 6-8 min, 7-21% acetonitrile from 8-18 min, 21-29% acetonitrile from 18-23 min, followed by a washing cycle; flow 1 ml min^−1^). Detection was performed with a photodiode array detector and peaks were integrated at 229 nm. We used the following response factors: a-GLS 2.0, iGLS 0.5 [85] for quantification of individual GLS.

### Quantitative ROS measurements

Quantitative ROS measurement were performed using the Amplex Red hydrogenperoxide/peroxidase assay kit (Molecular Probes) according to the manufacturer’s instructions (http://tools.invitrogen.com/content/sfs/manuals/mp 22188.pdf). ROS measurements were performed using the substrate carboxy-H_2_DFFDA (Molecular Probes) according to the manufacturer’s instructions (https://tools.invitrogen.com/content/sfs/ manuals/mp36103.pdf). The plant material was incubated in 20 μM carboxy-H_2_DFFDA prepared in KRPG buffer for 30 min in the dark. The fluorescence intensity was quantified with a fluorescence microplate reader (TECAN Infinite 200) with an excitation at 485 nm and emission at 530 nm. The reaction mixture without the substrate and plant material served as control.

### Quantitative reverse transcription-PCR Analysis

Total RNA was extracted using RNeasy Plant Mini kit with DNAse I treatment (Qiagen). cDNA was synthesised with the Omniscript cDNA synthesis kit (Qiagen) and 1 μg RNA. The oligonucleotide primers are given in Additional file 1: Table S2. The mRNA levels for each cDNA probe were normalized with respect to the *GAPDHC* message levels and expressed relative to the WT control [71]. Real-time quantitative RT-PCR was performed using the iCycler iQ real-time PCR detection system and iCycler software version 2.2 (Bio-Rad). For the amplification of the PCR products, iQ SYBR Supermix (Bio-Rad) was used according to the manufacturer’s instructions in a final volume of 23 μl. The iCycler was programmed to 95°C 2 min, 32× (95°C 30 s, 56°C 30 s, 72°C 30 s), 72°C 10 min followed by a melting curve program (55-95°C in increasing steps of 0.5°C). All reactions were repeated twice. The mRNA levels for each cDNA probe were normalized with respect to the *GAPDHC* message levels. Fold induction values were calculated with the ΔΔCP equation of Pfaffl [86]. The ratio of a target gene was calculated in the treated sample versus the untreated control in comparison to a reference gene.

## Abbreviations

ABA: Abscisic acid; aGLS: Aliphatic glucosinolates; [Ca^2+^]_cyt_: Cytosolic calcium; *cycam1*: Cytosolic calcium elevation mutant1; Chl: Chlorophyll; *cis*-12-OPDA: *cis*-12-oxo-phytodienoic acid; CWE: Cell wall extract; EMS: Ethyl methane sulfonate; EPM: Extract preparation from mycelia; EPS: Extract preparation from spores; GLS: Glucosinolates; iGLS: Indolic glucosinolates; JA: Jasmonic acid; JA-Ile: Jasmonyl-isoleucine; PA: Phsophatidic acid; PAMP: Pathogen-associated molecular pattern; ROS: Reactive oxygen species; SA: Salicylic acid; Tox: Toxin WT, wild-type.

## Competing interests

The authors declare that they have no competing interests.

## Author’s contributions

JMJ and RO designed and planned the research. JMJ performed the experiments. MR measured phytohormones, camalexin, and glucosinolates. JMJ analysed the data. JV and JG contributed to the discussion. JMJ and RO wrote the article. RO supervised the research. All authors read and approved the final manuscript.

## Supplementary Material

Additional file 1: Figure S1 Dose dependent increase of [Ca^2+^]_cyt_ elevation in Arabidopsis roots after treatment with *A. brassicae* PAMPs or toxin. **Figure S2.** Physical and chemical properties of CWE, EPM, EPS and toxin (Tox) from *A. brassicae*. **Figure S3.** Response of WT and *cycam* to CWE and EPM from *Rhizoctonia solani* (A, B), *Phytophthora parasitica* var. *nicotianae* (C, D), and the CWE from *Agrobacterium tumefaciens* (E). **Figure S4.** The *cycam1-1* and *cycam1-2* are more susceptible to *Rhizoctonia solani* and *Phytophthora parasitica* var. *nicotianae* infection. **Figure S5.** Photosynthetic parameters are impaired in *cycam* in response to different abiotic stress. **Figure S6.** Phytohormone regulated genes in WT and *cycam* seedlings. **Table S1.** Inhibition of [Ca^2+^]_cyt_ elevation induced by the *A. brassicae*-derived CWE, EPM, EPS and Tox preparations by staurosporine in WT roots. **Table S2.** Primer list for RT-PCR. Click here for file
