# Supplementary material for: An Arabidopsis mutant impaired in intracellular calcium elevation is sensitive to biotic and abiotic stress
Source: BMC Plant Biol. 2014 Jun 11;14:162. doi: 10.1186/1471-2229-14-162 (PMC4074868; doi:10.1186/1471-2229-14-162)
Supplement: Additional file 1: Figure S1 — Dose dependent increase of [Ca2+]cyt elevation in Arabidopsis roots after treatment with A. brassicae PAMPs or toxin. Figure S2. Physical and chemical properties of CWE, EPM, EPS and toxin (Tox) from A. brassicae. Figure S3. Response of WT and cycam to CWE and EPM from Rhizoctonia solani (A, B), Phytophthora parasitica var. nicotianae (C, D), and the CWE from Agrobacterium tumefaciens (E). Figure S4. The cycam1-1 and cycam1-2 are more susceptible to Rhizoctonia solani and Phytophthora parasitica var. nicotianae infection. Figure S5. Photosynthetic parameters are impaired in cycam in response to different abiotic stress. Figure S6. Phytohormone regulated genes in WT and cycam seedlings. Table S1. Inhibition of [Ca2+]cyt elevation induced by the A. brassicae-derived CWE, EPM, EPS and Tox preparations by staurosporine in WT roots. Table S2. Primer list for RT-PCR. [file 1471-2229-14-162-S1.doc]

**Additional file 1: Figure S1.**

**Dose dependent increase of** **[Ca^2+^]_cyt_ elevation in Arabidopsis roots after treatment with *A. brassicae* PAMPs or toxin.** [Ca^2+^]_cyt_ elevation was measured in the roots of 18 day-old wild-type seedlings grown on Hoagland medium under long-day conditions. The roots were incubated overnight in 7.5 μM coelenterazine and treated with different concentrations of the CWE **(A)**, EPM **(B)**, EPS **(C)** or toxin **(D)**. [Ca^2+^]_cyt_ elevation increases with increase in the dose of PAMPs and toxin from *A. brassicae.* The dilutions were made with sterile water. The control treatment was performed with sterile H_2_O which gave background readings. All curves represent means of four independent experiments with eight replications in each experiment.

**Time [min]**

**Time [min]**


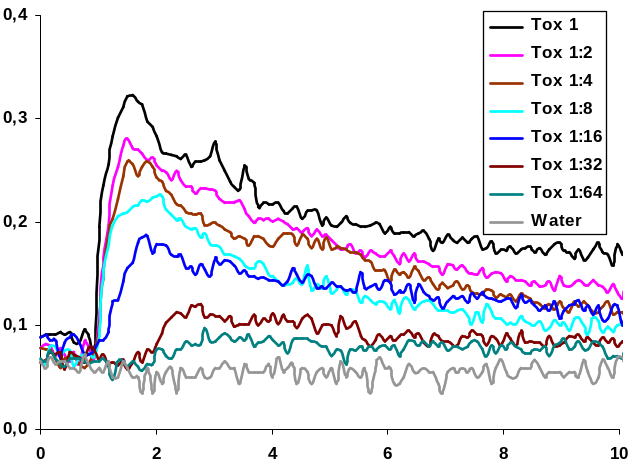


**[Ca]^2+^_cyt_ (μM)**

**[Ca]^2+^_cyt_ (μM)**

**D**

**B**

**C**

**A**

**Additional file 1: Figure S2.**

**Physical and chemical properties of CWE, EPM, EPS and toxin (Tox) from *A. brassicae*.** Heat treatment, extraction with ethyl acetate, precipitation with methanol and separation into different molecular size fractions of CWE, EPM, EPS and toxin were performed as described below. The [Ca^2+^]_cyt_ elevation peak values are not changed after autoclaving the CWE, EPM, EPS or toxin preparations at 121^0^C for 20 min **(A)**. After extraction with equal volume of ethyl acetate for 1 h, the organic and aqueous phases were concentrated, dried and the lyophilised powder was dissolved in sterile water to its original volume. Only the aqueous phase was active. The organic phase did not induce [Ca^2+^]_cyt_ elevation in roots **(B)**. After precipitation with 80% methanol (ppt) for 10 min, only the supernatant (supt) of the CWE, EPM and EPS preparations and not the resuspended precipitates induced [Ca^2+^]_cyt_ elevation in roots. Both supernatant and precipitate of the toxin preparation were active **(C)**. After size separation into > 10, 3-10 and < 3 kilo-Dalton (kDa) fractions, the activity was found in the < 3 kDa fractions. The > 10 and 3-10 kDa fractions did not show [Ca^2+^]_cyt_ elevation activities in roots **(D)**. Data are means of three independent experiments with eight replications in each experiment.

**Methods:**

**Fractionation of the CWE, EPM, EPS and the Tox preparations from *A. brassicae***

Fractionation of the *A. brassicae* exudate fractions was carried out on Roti Spin Mini-10 or Roti Spin Mini-3 centrifugal filter devices with 10 and 3 kDa molecular weight cut-offs (Roth, Germany) according to the protocol of the manufacturer. The membranes were sanitised with 70% ethanol and thrice with sterile H_2_O; pre-rinsed with 50 mM NaOH and then thrice with sterile H_2_O. Extraction with ethyl acetate was performed by adding an equal volume of ethyl acetate to the [Ca^2+^]_cyt_-inducing fractions. After 30 min of shaking, the sample was centrifuged at 6,000 g for 5 min, and the organic phase was collected. The extraction was repeated. The organic and aqueous phases were evaporated to dryness in a vacuum centrifuge and bioassayed for [Ca^2+^]_cyt_-inducing activity after re-suspension in sterile H_2_O to the initial volume (Navazio et al., 2007). The Ca^2+^-inducing fractions were also extracted into 80% methanol. After mixing for 2 x 30 min, the sample was centrifuged at 6,000 *g* for 5 min and the supernatant was collected. The extraction was repeated. The supernatants were pooled and evaporated to dryness in a vacuum centrifuge and bioassayed for [Ca^2+^]_cyt_ elevation after adding sterile H_2_O. The precipitate was re-suspended in sterile H_2_O to test for its [Ca^2+^]_cyt_-inducing activity.

**Navazio, L., Moscatiello, R., Genre, A., Novero, M., Baldan, B., Bonfante, P., and Mariani, P.** (2007). A diffusible signal from arbuscular mycorrhizal fungi elicits a transient cytosolic calcium elevation in host plant cells. Plant Physiol. **144**, 673-681.


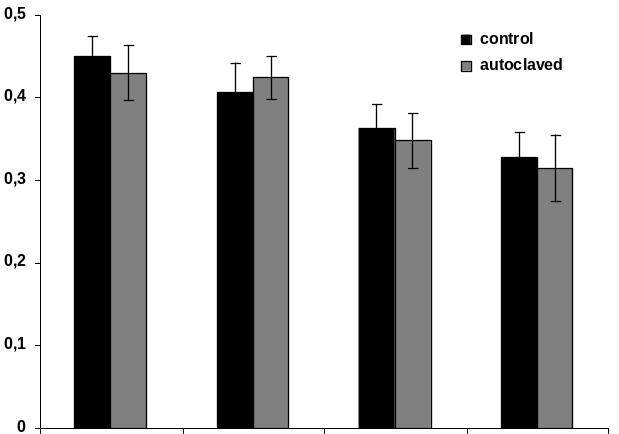

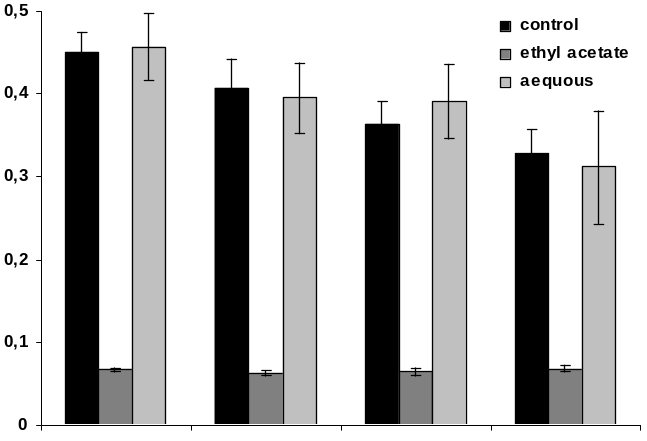

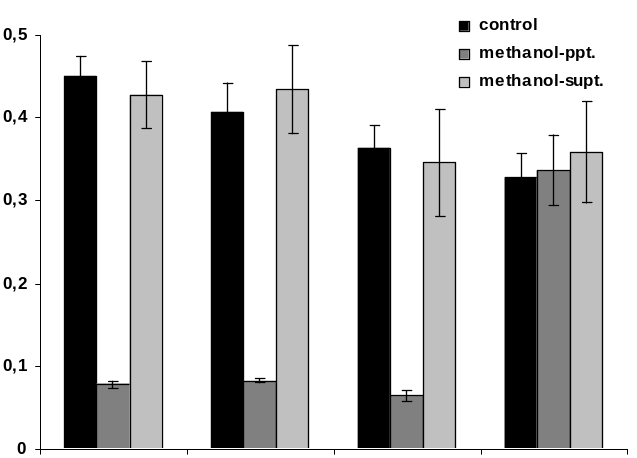

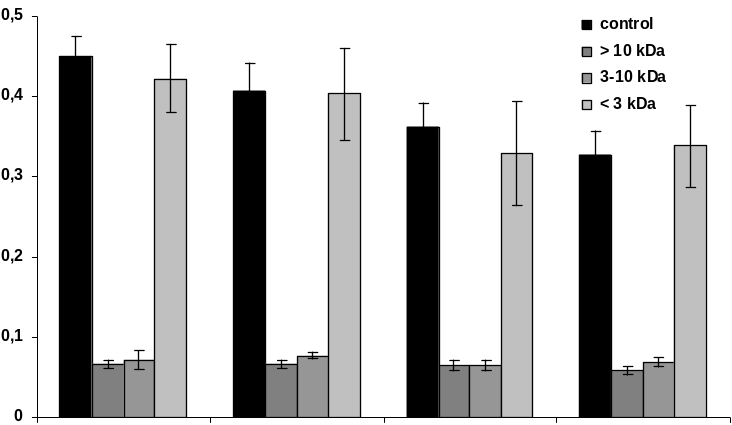


**CWE EPM EPS Tox CWE EPM EPS Tox**

**B**

**C**

**D**

**[Ca]^2+^_cyt_ (μM)**

**[Ca]^2+^_cyt_ (μM)**

**A**

**Additional file 1: Figure S3.**

**Response of WT and *cycam* to CWE and EPM from *Rhizoctonia solani* (A, B), *Phytophthora parasitica* var. *nicotianae* (C, D), and the CWE from *Agrobacterium tumefaciens* (E).** The roots of WT, *cycam1-1* or *cycam1-2* seedlings were challenged with 50 μl of CWE **(A, C, E)** or EPM **(B, D)** of the microbes. The roots of *cycam1-1* and *cycam1-2* did not respond to the CWEs and EPMs of the microbes, the response to the *A. tumefaciens* CWE was reduced. WT aequorin roots served as control. All curves represent mean of four independent experiments with eight replications in each experiment.

**Methods:**

*R. solani* (FSU-1137), *P. parasitica* (FSU-746) and *M. hyalina* (FSU-509) were obtained from Jena Microbial Resource Centre, Jena, Germany. These fungi were grown on potato dextrose agar (PDA) medium (pH 6.5-6.7) at 20 ± 1°C in a temperature-controlled chamber under 12/12 h light/dark and 75% relative humidity for 2 weeks. To maintain the virulence, the fungi were inoculated to Arabidopsis seedlings and re-isolated from the infected tissues periodically (Johnson et al., 2013).

For the *A. tumefaciens* CWE preparation, the cells grown in yeast extract medium for 48 h were harvested by centrifugation and washed 5 times with distilled water before homogenization with a Warring blender. The residual procedure was identical to that described for the fungi in the Methods and Material Section.

**Johnson, J.M., Sherameti, I., Nongbri P.L. and Oelmüller, R.** (2013). Standardized conditions to study beneficial and nonbeneficial traits in the *Piriformospora indica*/ *Arabidopsis thaliana* interaction. In. *Piriformospora indica*: Sebacinales and their biotechnological applications; Soil Biology. **33**, 325-343. A. Varma et al. (eds.), Springer-Verlag Berlin Heidelberg Germany.


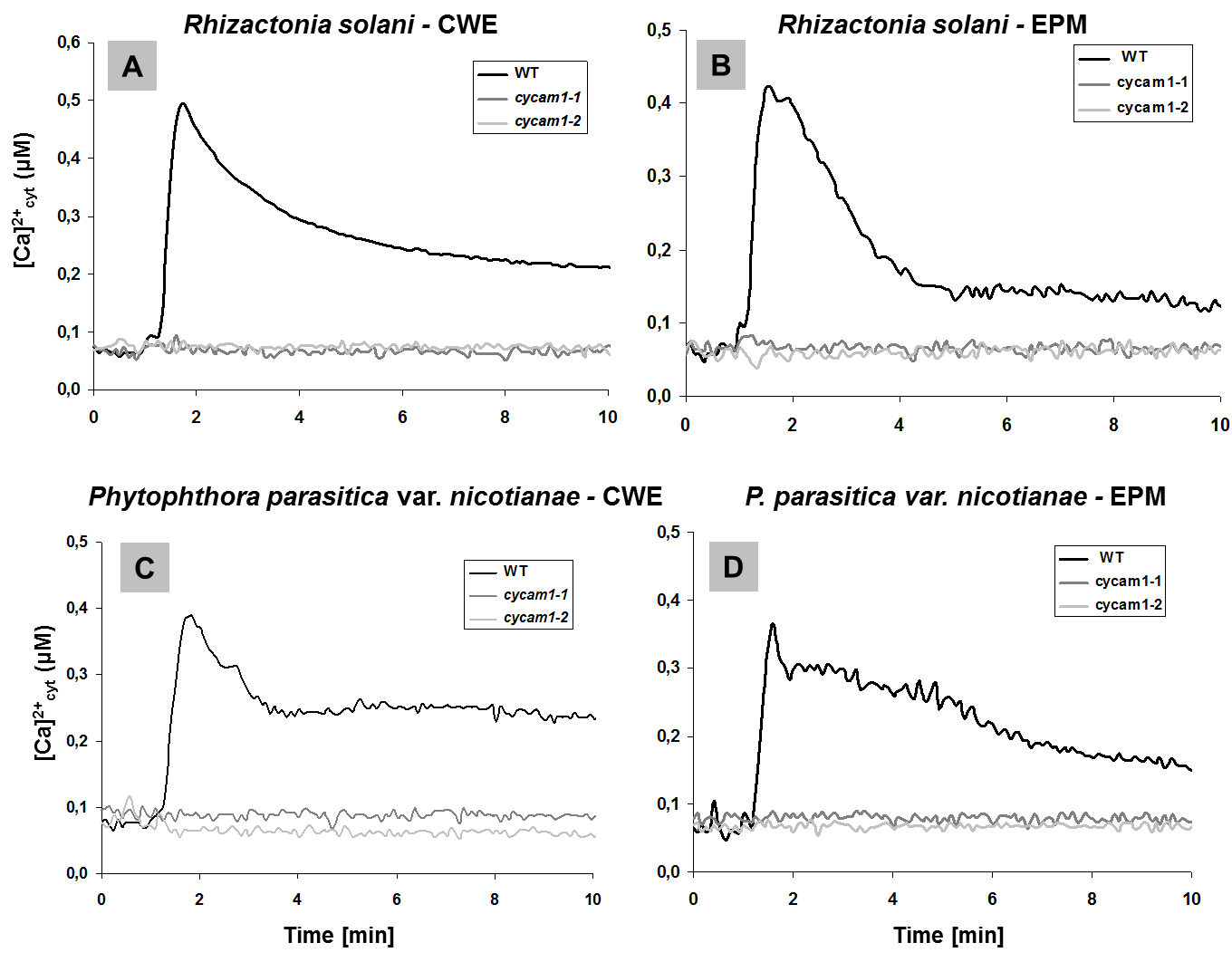


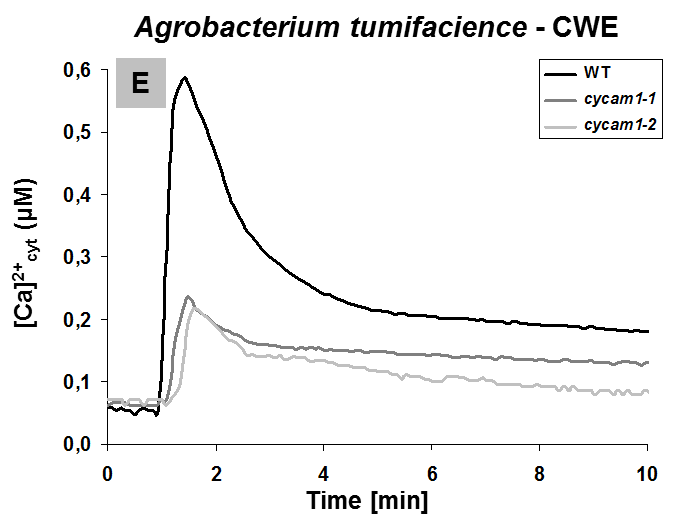


**Additional file 1: Figure S4.**

**The *cycam1-1* and *cycam1-2* are more susceptible to *Rhizoctonia solani* and *Phytophthora parasitica* var. *nicotianae* infection.** 14-day old *cycam1* and WT seedlings grown under long-day conditions were exposed to a 7-day old fungal lawn (in PDA) of *R. solani* and *P. parasitica* var. *nicotianae* and incubated under long-day conditions for 2 weeks. Seedlings kept on PDA alone served as control. Representative pictures are shown from three independent experiments with 10 replications containing 30 seedlings per line in each experiment.

**Methods:**

**Growth of Arabidopsis seedlings on a *R. solani* and *P. parasitica* var. *nicotianae* lawn**

14-day-old WT and *cycam* seedlings were directly transferred from MS medium to a plate with a fungal lawn (Vahabi et al., 2013). Control seedlings were transferred to Kaefer medium without the fungus. The fungal lawns of *R. solani* and *P. parasitica* var. *nicotianae* were obtained by placing their fungal plugs on PDA medium and the fungi were allowed to grow for 7 days at 22°C with 12h/12h light/dark cycle before transferring the seedlings to the plate. The plates were incubated for 2 weeks at 20°C under long-day conditions with a light intensity of 80 µmol m^-2^ sec^-1^ from above.

**Vahabi K, Camehl I, Sherameti I, and Oelmüller R.** (2013). [Growth of Arabidopsis seedlings on high fungal doses of *Piriformospora indica* has little effect on plant performance, stress, and defense gene expression in spite of elevated jasmonic acid and jasmonic acid-isoleucine levels in the roots.](http://www.ncbi.nlm.nih.gov/pubmed/24047645) Plant Signal Behav. Sep 18;8(11). pii: e26301.


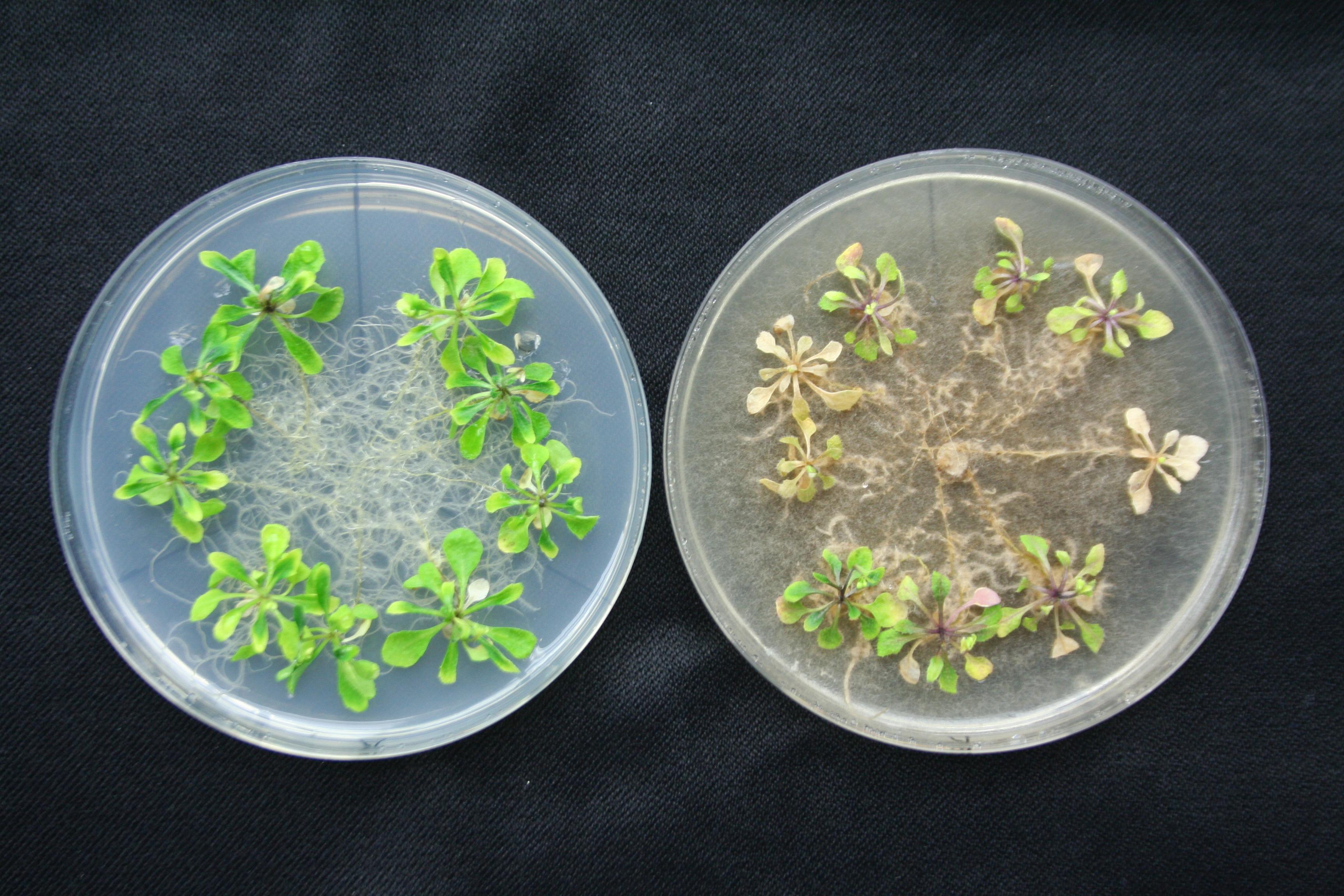

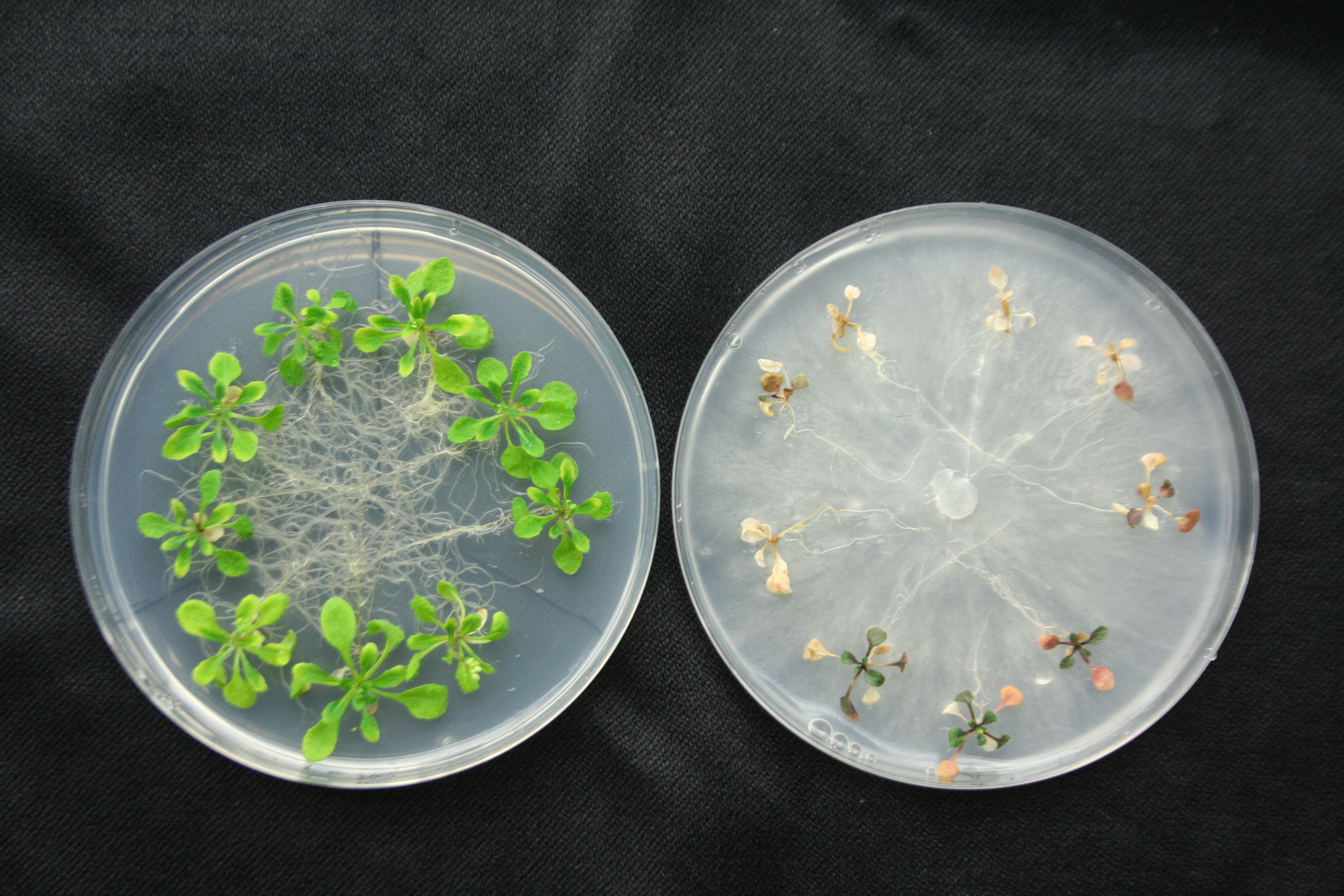


***cycam1-1 cycam1-2 cycam1-1 cycam1-2***

**WT WT**

***cycam1-1 cycam1-2 cycam1-1 cycam1-2***

**WT WT**

**control *P. parasitica var. nicotianae***

**control*R. solani***

**Additional file 1: Figure S5.**

**Photosynthetic parameters are impaired in *cycam* in response to different abiotic stress.**

Maximum quantum yield of PSII (F_v_/F_m_), photochemical quenching (qP) (**A**), quantum yield of PSII (Φ_PSII_) and non-photochemical quenching (NPQ) (**B**) were determined for 14-day old WT, *cycam1-1* and *cycam1-2* seedlings grown on MS medium amended with 100 nM ABA, 100 mM NaCl or 350 mM mannitol under long-day conditions. For experimental details, cf. Vahabi et al. (2013). Data are means ± SEs from 5 independent experiments with > 40 seedlings per treatment in each experiment.

**Vahabi K, Camehl I, Sherameti I, and Oelmüller R.** (2013). [Growth of Arabidopsis seedlings on high fungal doses of *Piriformospora indica* has little effect on plant performance, stress, and defense gene expression in spite of elevated jasmonic acid and jasmonic acid-isoleucine levels in the roots.](http://www.ncbi.nlm.nih.gov/pubmed/24047645) Plant Signal Behav. Sep 18;8(11). pii: e26301.

**A**

**F_v_/F_m_ qP**

**B**

**Φ_PSII_  NPQ**

**Additional file 1: Figure S6**.

**Phytohormone regulated genes in WT and *cycam* seedlings.** mRNA levels of SA- **(A)**, ABA- **(B)** and JA- **(C)** biosynthesis and responsive genes in the leaves 2 days after infection with *A. brassicae* spores (+ *A. brassicae*) or mock-treatment with sterile water (- *A. brassicae*). The leaves of 12-day old WT, *cycam1-1* and *cycam1-2* seedlings grown on MS under LD conditions were inoculated with a fungal spore suspension containing 10^4^ to 10^5^ cfu ml^-1^ and incubated under LD conditions for 3 additional days. The abbreviations of the genes and annotation numbers are given. The mRNA levels for each cDNA probe were normalized with respect to the plant GAPDHC message levels. The mRNA levels in the mock-treated WT seedlings were set as 1.0 and the other values expressed relative to it. The values are means ± SEs of four independent RT-PCR experiments with three replications in each experiment.


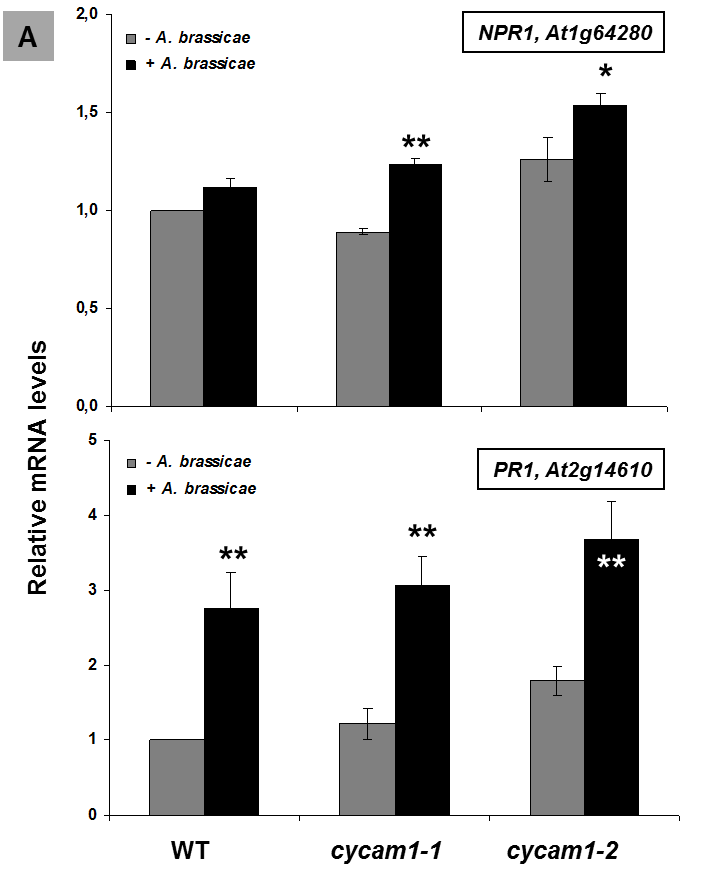


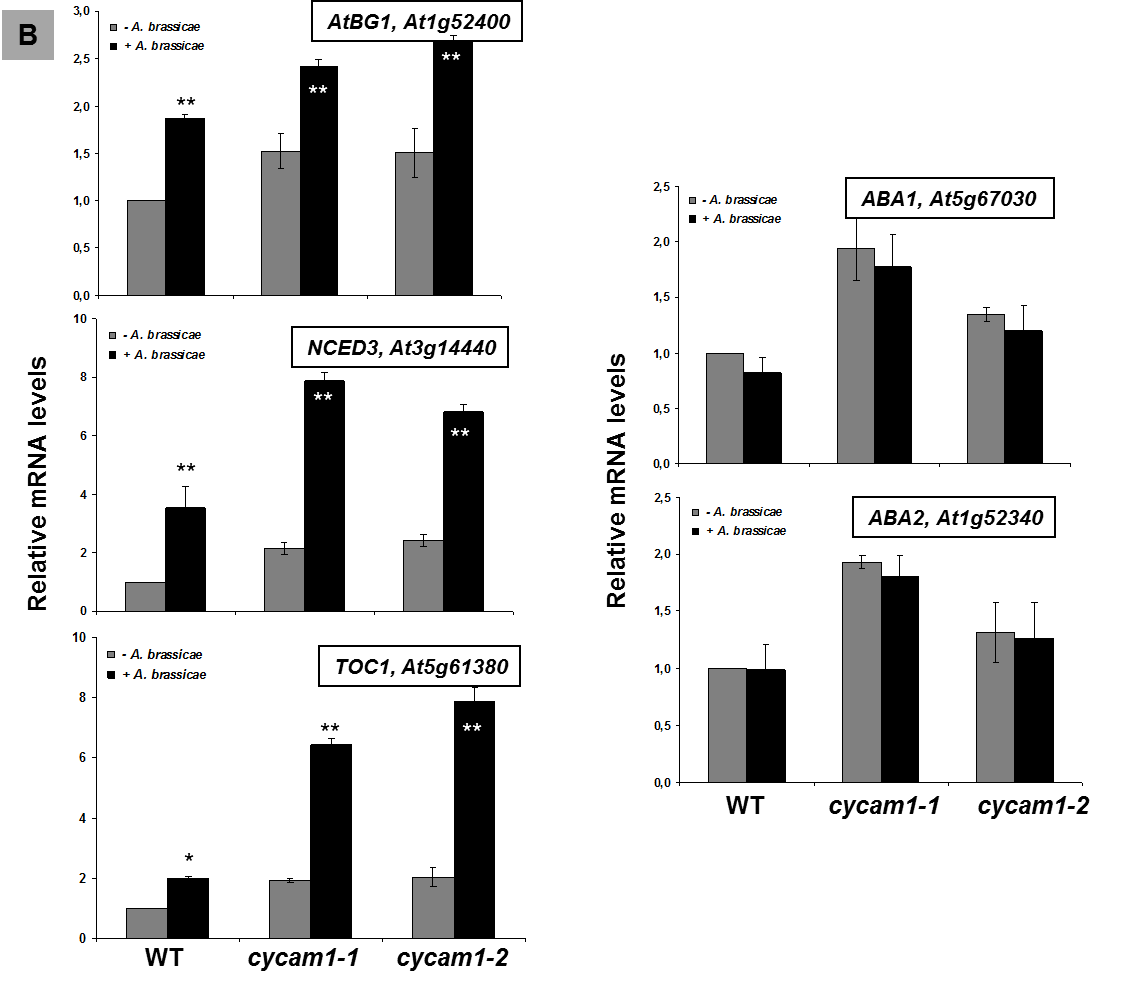


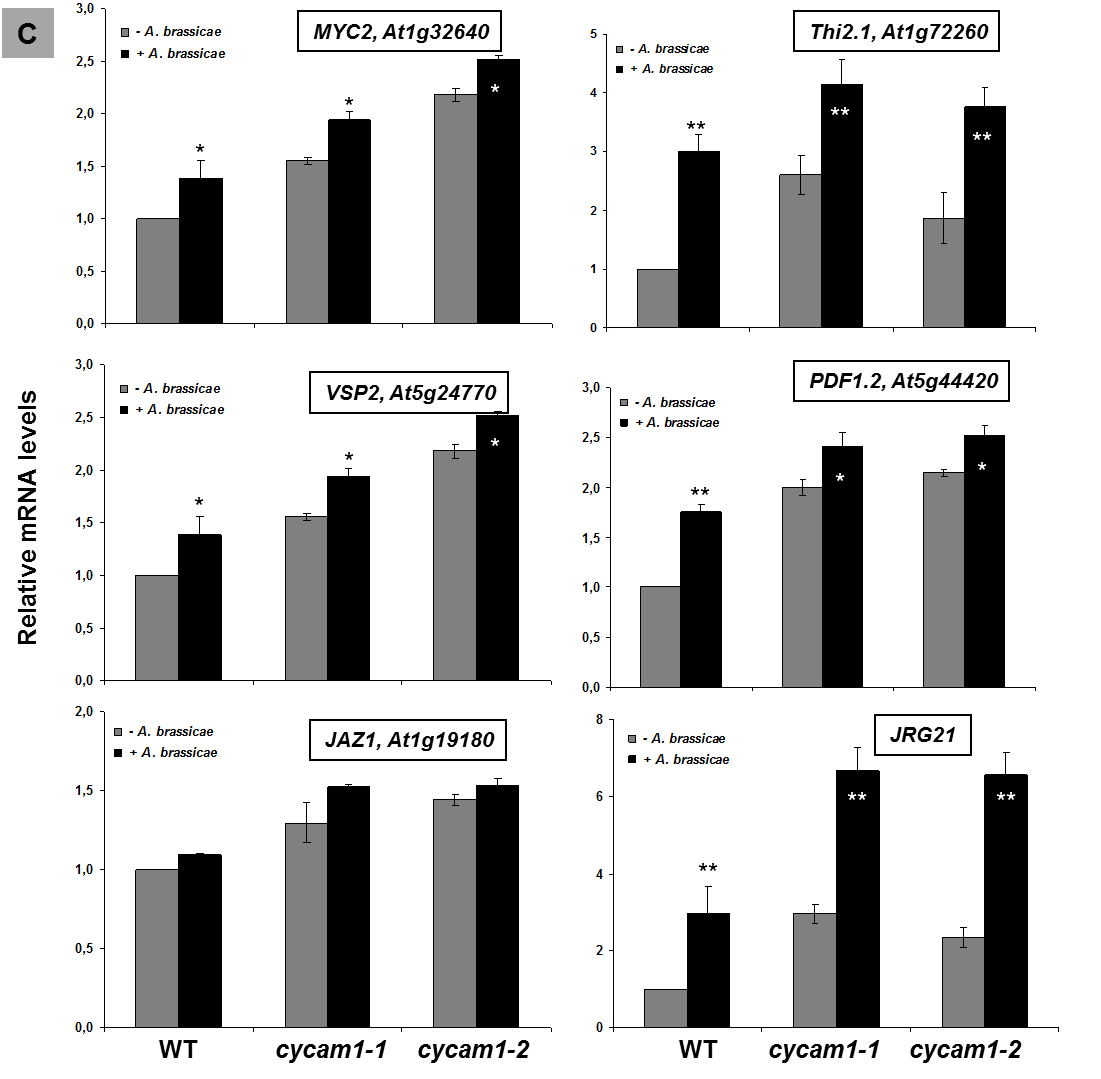


**Additional file 1: Table S1.**

**Inhibition of [Ca^2+^]_cyt_ elevation induced by the *A. brassicae*-derived CWE, EPM, EPS and Tox preparations by staurosporine in WT roots.** Roots of 18 day-old seedlings were dissected and incubated overnight in 7.5 μM coelenterazine. The roots were incubated with staurosporine (5 μM in 0.1% DMSO) or 0.1% DMSO (mock treatment) for 1 h after removing the coelenterazine solution. Then, [Ca^2+^]_cyt_ elevation was induced by 50 μl of CWE, EPM, EPS or Tox preparation. The peak values for WT roots are given. All values represent means of three independent experiments with sixteen replications in each experiment.

Staurosporine was purchased from LC Laboratories MA 01801, USA. A stock solution was prepared in 0.1% DMSO.

| **Treatment** | **no staurosporine**  **μM [Ca^2+^]_cyt_** | **+ staurosporine**  **μM [Ca^2+^]_cyt_** |
| --- | --- | --- |
| H_2_O or 0,1% DMSO | 0,058 ± 0,002 | 0,112 ± 0,006 |
| CWE | 0,282 ± 0,009 | 0,122 ± 0,012 |
| H_2_O or 0,1% DMSO | 0,053 ± 0,003 | 0,098 ± 0,008 |
| EPM | 0,249 ± 0,016 | 0,096 ± 0,007 |
| H_2_O or 0,1% DMSO | 0,063 ± 0,004 | 0,128 ± 0,013 |
| EPS | 0,277 ± 0,016 | 0,122 ± 0,015 |
| H_2_O or 0,1% DMSO | 0,068 ± 0,004 | 0,096± 0,003 |
| Tox | 0,279 ± 0,014 | 0,135 ± 0,002 |

**Additional file 1: Table S2.**

**Primer list for RT-PCR.**

| **AGI** | **Gene Name** | **Forward primer** | **Reverse primer** |
| --- | --- | --- | --- |
| At3g55970 | *JRG21* | AGTGGTGAACCACGGGATGAGT | GGCAATGAAGAGGCAAGGAAGG |
| At1g32640 | *MYC2* | CGGAGATCGAGTTCGCCGCC | AATCCCGCACCGCAAGCGAA |
| At5g24770 | *VSP2* | ACGACTCCAAAACCGTGTGCAA | CGGGTCGGTCTTCTCTGTTCCGT |
| At1g19180 | *JAZ1* | GGCGAGCAAAGGCACCGCTA | TCCAAGAACCGGTGAAGTGAAGC |
| At1g72260 | *Thi2.1* | CGCCATTCTCGAAAACTCAGCTGA | GTTTAGGCGGCCCAGGTGGG |
| At5g44420 | *PDF1.2* | CTGCTTTCGACGCACCGGCA | GTTGCATGATCCATGTTTGGCTCCT |
| At1g64280 | *NPR1* | ACATAGTCGGCCTTTGAGAGA | AAGGTGCAAGTGCATCAGAAG |
| At2g14610 | *PR1* | GTGCAATGGAGTTTGTGGTC | TCACATAATTCCCACGAGGA |
| At1g52400 | *AtBG1* | TTACTATACTTCAGTGTTTGCAAAAG | CTAGAGTTCTTCCCTCAGCTTG |
| At3g14440 | *NCED3* | ATGGCTTCTTTCACGGCAAC | GTCTCCGTCGAAGAAGTG |
| At5g61380 | *TOC1* | GCTAGGACTTGCTGAGAAGA | TCCCTCTACTTCTGTGTGCT |
| At5g67030 | *ABA1* | ATGGGTTCAACTCCGTTTTGC | CATACCAAGTACCAGAGATACC |
| At1g52340 | *ABA2* | ATGTCAACGAACACTGAATCTTCTTC | GCATGCTTGGAACCAACATAAG |
| At5g61420 | *Myb28* | GTGGTCGGTCATAGCGAGAC | GCGAGTCTGAGTCGGTGTCA |
| At5g07690 | *Myb29* | TACAACGGTCGTCTACCACA | TCATCCGGGTTTGAGTCATA |
| At3g19710 | *BCAT4* | CTTCAAGCCGGAGCTGACAG | CAGCCCTGGCGGTCAATCT |
| At4g39950 | *CYP79B2* | GCCGGATATCACATCCCTAA | TCCGGTTTAAAGCAAAGTGG |
| At2g22330 | *CYP79B3* | CGTGGCACTCTCTGATACGA | CAGACCAAACCTTGGGGTTA |
| At2g30770 | *CYP71A13* | GGGTAGAGGCTGGACCAAAT | ACAACCGAAGATGGAAATGC |
| At3g26830 | *PAD3* | GGTACGGGATAAATCTCTATGA | AGATACAGTCGATGAACCTAC |
| At4g34410 | *RRTF1* | ACAGTGATAAGCGCGGGAAT | TCCACAAAGGGGAAGTTGAG |
| At1g10585 | *bHLH TF* | GGATCGAAGGATGCGCATG | CCCGCGAACGAATACTGAG |
| At3g55970 | *ZF (CCCH)* | AGTGGTGAACCACGGGATGAGT | GGCAATGAAGAGGCAAGGAAGG |
| At1g63040 | *AP2/EREBP* | ACGTGTCTGGCTTGGGAC | TATCCGCTAGCAAACCAGGC |
| At4g11210 | *DSR* | GTTTCGTCCTCCACCTCCAT | ACCACTGTCCAAGCGATCC |
| At1g21100 | *OMT* | TCGTCTACTCGCTAGCTACTCC | ATACCACCATGTGCGCGAC |
| At3g25250 | *OXI1* | GTGAGTTTCGAGCAAGGAGT | GCCGCGTAAAATCTGATAATC |
| At3g49620 | *DIN11* | ACATGGGGACATCGGAAAGG | TCAGTGTGAGCTCCACATCC |
| At4g25100 | *FSD1* | TCACTGGGGAAAACATCACA | GGATTCACAGCATTGGGAGT |
| At2g28190 | *CSD2* | TTCCTCCTTCCTCCAATCCT | AGGGTTGAAATGTGGTCCTA |
| At1g07890 | *APX1* | CCACTCGCATTTCTCCAGAT | TCGAAAGTTCCAGCAGAGTG |
| At1g19570 | *DHAR5* | CAGTCACCCACTTGTCGTCG | GTCCGTTCAGCCAACGGGC |
| At3g09940 | *MDAR2* | CCTCTCCATCTGAGTTGGTGC | GCGGCGGTTTCTTAGGGC |
|  | *AbreATR1* | ACCCGCATTCCTCGCCAAA | AAGTCAAGGATTGTGTCGAGCTT |
| At3g18780 | *Actin 2* | ggaatccacgagacaaccta | ATCTTCATGCTGCTTGGTGC |
| At3g04120 | *GAPDHC* | CTAGAGTTGTTCTCCAGAGGGAC | CAAGGCAGTTAGTGGTGCAGCTA |
